# Unsupervised analysis reveals two molecular subgroups of serous ovarian cancer with distinct gene expression profiles and survival

**DOI:** 10.1007/s00432-016-2147-y

**Published:** 2016-03-30

**Authors:** Katarzyna M. Lisowska, Magdalena Olbryt, Sebastian Student, Katarzyna A. Kujawa, Alexander J. Cortez, Krzysztof Simek, Agnieszka Dansonka-Mieszkowska, Iwona K. Rzepecka, Patrycja Tudrej, Jolanta Kupryjańczyk

**Affiliations:** Center for Translational Research and Molecular Biology of Cancer, Maria Skłodowska-Curie Memorial Cancer Center and Institute of Oncology, Gliwice Branch, Gliwice, Poland; Department of Automatic Control, Silesian Technical University, Gliwice, Poland; Department of Pathology, Maria Skłodowska-Curie Memorial Cancer Center and Institute of Oncology, Warsaw, Poland

**Keywords:** Ovarian cancer, Gene expression analysis, Prognostic biomarkers, Singular value decomposition (SVD), Dermatan sulfate proteoglycan 3 (DSPG3), Lysyl oxidase (LOX)

## Abstract

**Purpose:**

Ovarian cancer is typically diagnosed at late stages, and thus, patients’ prognosis is poor. Improvement in treatment outcomes depends, at least partly, on better understanding of ovarian cancer biology and finding new molecular markers and therapeutic targets.

**Methods:**

An unsupervised method of data analysis, singular value decomposition, was applied to analyze microarray data from 101 ovarian cancer samples; then, selected genes were validated by quantitative PCR.

**Results:**

We found that the major factor influencing gene expression in ovarian cancer was tumor histological type. The next major source of variability was traced to a set of genes mainly associated with extracellular matrix, cell motility, adhesion, and immunological response. Hierarchical clustering based on the expression of these genes revealed two clusters of ovarian cancers with different molecular profiles and distinct overall survival (OS). Patients with higher expression of these genes had shorter OS than those with lower expression. The two clusters did not derive from high- versus low-grade serous carcinomas and were unrelated to histological (ovarian vs. fallopian) origin. Interestingly, there was considerable overlap between identified prognostic signature and a recently described invasion-associated signature related to stromal desmoplastic reaction. Several genes from this signature were validated by quantitative PCR; two of them—*DSPG3* and *LOX*—were validated both in the initial and independent sets of samples and were significantly associated with OS and disease-free survival.

**Conclusions:**

We distinguished two molecular subgroups of serous ovarian cancers characterized by distinct OS. Among differentially expressed genes, some may potentially be used as prognostic markers. In our opinion, unsupervised methods of microarray data analysis are more effective than supervised methods in identifying intrinsic, biologically sound sources of variability. Moreover, as histological type of the tumor is the greatest source of variability in ovarian cancer and may interfere with analyses of other features, it seems reasonable to use histologically homogeneous groups of tumors in microarray experiments.

**Electronic supplementary material:**

The online version of this article (doi:10.1007/s00432-016-2147-y) contains supplementary material, which is available to authorized users.

## Introduction

In most gene expression studies, data analysis is carried out using so-called supervised methods that rely on the arbitrary division of analyzed samples into classes that are then compared in order to identify differentially regulated genes and molecular pathways. This approach works well when performing simple in vitro experiments with well-defined experimental variables (e.g., Fiszer-Kierzkowska et al. 2011; Olbryt et al. 2014). However, human tumor samples are more complex, and the major drawback of supervised methods is that stratification of these samples using arbitrarily chosen criteria may not accurately reflect the true biological checkpoints underlying the feature of interest. In addition, criteria for classifying the same feature can vary between studies. These methodological issues are rarely acknowledged, although they may be among the major reasons why microarray studies in cancer research have low reproducibility and fail to find new molecular markers.

In our previous study, using similar set of ovarian cancer samples, we carried out supervised analyses in relation to several clinicopathological features in order to delineate the molecular background of ovarian cancer chemoresistance and identify biomarkers suitable for predicting patient prognosis. However, only four of 18 genes that were selected as possible markers for chemotherapy response and survival were validated by quantitative PCR in the initial set of samples (Lisowska et al. 2014), and only one gene—*cytoplasmic linker*-*associated protein 1*—was validated in an independent set of ovarian tumors with respect to overall survival (OS) and disease-free survival (DFS). In addition, the majority of significant genes identified in these previous supervised analyses were not confirmed in other studies, as revealed by literature search.

In the present study, we analyzed the microarray data from 101 ovarian cancer samples by singular value decomposition (SVD), an unsupervised method of data analysis that allows to reveal the major sources of variability in a complex dataset. In contrast to supervised methods, in SVD, no prior assumptions are made (i.e., there are no arbitrarily defined classes) and data can organize themselves. In this way, SVD enables class detection in analyzed dataset, e.g., identification of novel subgroups of cancers or patients and/or co-expressed genes.

This approach showed that the greatest source of variability in our dataset was attributable to the histological type of ovarian cancer. Interestingly, it appeared that the next major source of variability was linked to patients’ OS. The genes associated with the latter were mostly related to the regulation of the extracellular matrix (ECM), cell motility, adhesion, and immunological response. Patients with higher expression of these genes had shorter OS than those with lower expression. A similar gene set was previously detected in a computational study of microarray data derived from several types of cancer (Kim et al. 2010); these authors postulated that this signature is acquired during molecular evolution of the cancer during progression from lower to higher stages and results from tumor infiltration by cancer-associated fibroblasts (CAFs). However, we present evidence that this signature may be expressed by ovarian cancer cells themselves.

## Materials and methods

### Clinical samples

Surgical samples were obtained during primary surgery, then snap-frozen in liquid nitrogen and stored at −80 °C. The tissue samples were collected at the Maria Skłodowska-Curie Memorial Cancer Center and Institute of Oncology in Warsaw, Poland. Only samples from patients without neoadjuvant chemotherapy were used in this study as chemotherapy may seriously affect gene expression profile. Tissue samples with stromal cell contamination level lower than 15 % were selected from a larger collection of tumors.

Initially, we analyzed 101 ovarian cancer specimens: 74 serous, 12 endometrioid, 9 clear cell, and 6 undifferentiated. Patients were diagnosed at FIGO stages II-IV. The tumors were graded in a four-grade scale, according to the criteria given in Barber et al. (1975). All these tumors were tested for somatic p53 mutation and majority of them were mutated (64 samples with mutation and 8 without) (Dansonka-Mieszkowska et al. 2006). The patients were also tested for BRCA1 gene mutation and 18 patients from this group had hereditary BRCA1 mutation, one patient had somatic BRCA1 mutation, while 54 patients had no mutation (Rzepecka et al. 2012). These and other data are given in Table 1.

**Table 1:** Characteristics of patients and tumor samples analyzed by microarray

Characteristics (total no.)		Numbers of samples							
Histology	(101)	Serous	74	Endometrioid	12	Clear cell	9	Undifferentiated	6
CHT response	(72)	CR	48	PR	14	SD	3	P	7
Platinum sensitivity^a^	(72)	Highly sensitive	12	Moderately sensitive	27	Resistant	33		
FIGO stage	(72)	FIGO II	3	FIGO III	59	FIGO IV	10		
Tumor grade	(77)	G2	10	G3	48	G4	19		
Residual tumor	(72)	R0	15	R1	36	R2	21		
*BRCA1* mutation	(72)	Mutation	19	No mutation	53				
*TP53* mutation	(72)	Mutation	64	No mutation	8				

*BRCA1* breast cancer 1, *CHT* chemotherapy response, described as clinical status of the patient after first-line treatment, *CR* complete remission, *FIGO* Federation of Gynecology and Obstetrics, *G2*–*G4* tumor grades 2–4, *P* progression, *PR* partial remission, *R0* residual tumor <1 cm, *R1* residual tumor between 1 and 5 cm, *R2* residual tumor >5 cm, *SD* stable disease, *TP53* tumor protein 53

^a^Tumors were classified as highly sensitive for DFS > 732 days, moderately sensitive for 732 days > DFS > 180 days, and resistant for DFS < 180 days

More in-depth analyses were done using only serous and undifferentiated samples with complete data concerning overall survival (OS) and disease-free survival (DFS). There were 68 serous and 4 undifferentiated tumors (Table 2).

**Table 2:** Distribution of the features for high-grade serous ovarian carcinomas in two clusters of serous and undifferentiated cancers with distinct overall survival (OS)

Feature	Cluster 2 (short OS)		Cluster 1 (long OS)		Fisher’s exact test *p* value
	No. of samples	% of samples	No. of samples	% of samples	
Grade 2	1	1.4	7	9.7	0.42
Grades 3 + 4	21	29.2	43	59.7	
*p53* mutation	20	27.8	44	61.1	1.0
*p53* no mutation	2	2.8	6	8.3	
*BRCA1* mutation	4	5.6	15	20.8	0.39
*BRCA1* no mutation	18	25	35	48.6	
Lower advanced stage (FIGO IIB–IIIB)	2	2.8	12	16.6	0.20
Higher advanced stage (FIGO IIIC–IV)	20	27.8	38	52.8	
Total	22	30.5	50	69.5	

*BRCA1* breast cancer 1, *FIGO* International Federation of Gynecology and Obstetrics

### RNA isolation

Total RNA was isolated from 3 to 5 sections (20 µm thick) of frozen tumor using RNeasy Mini Kit (Qiagen) with simultaneous on column DNase I digestion. RNA purity and concentration were estimated with ND-1000 spectrophotometer (NanoDrop Technologies). RNA quality was assessed using Agilent platform: RNA 6000 Nano LabChip Kit, RNA Integrity Number software, and the Agilent 2100 Bioanalyzer **(**Agilent Technologies). The samples with RIN values above 7 (full range 0–10) were accepted for further processing.

### Oligonucleotide microarrays

We used HG U133 Plus 2.0 GeneChip oligonucleotide arrays (Affymetrix). Total RNA (8 μg) was used for synthesis of double-stranded cDNA. Biotinylated cRNA was synthesized with the BioArray High Yield RNA Transcript Labeling Kit (Enzo Diagnostics). Both cDNA and cRNA were purified with GeneChip Sample Cleanup Module (Affymetrix). cRNA (16 μg) was fragmented and hybridized to the microarray for 16 h at 45 °C. The microarrays were stained, washed, and subsequently scanned with GeneChip Scanner 3000 (Affymetrix). Data were acquired using GCOS 1.2 software (Affymetrix). The preprocessing was performed by robust multi-array analysis (RMA, Bioconductor). Raw preprocessed data together with detailed descriptions of the samples are available at Gene Expression Omnibus repository under accession no Series GSE63885.

### Reverse transcription and quantitative PCR

Half a μg of total RNA was taken for cDNA synthesis using Omniscript RT Kit (Qiagen), random primers (4 μM, Sigma-Aldrich), oligo(dT) primer (1 μM, QBiogene Inc.), and RNase inhibitor (10 U, Fermentas). The reaction was performed in 20 µl of total volume, according to manufacturer’s protocol, using thermocycler UNO II (Biometra). The cDNA was diluted tenfold and a 5 μl aliquot was taken for real-time PCR performed using Taqman 2x PCR Master Mix (Roche), Exiqon probe (100 nM) and appropriate primers (200 nM each; Supplementary Table 1) designed using dedicated software from the Roche Web site. The reaction was carried out using ABI PRISM 7700 Sequence Detection System (Applied Biosystems) at the following conditions: 2 min at 50 °C, 10 min at 95 °C, 40 cycles of 15 s at 95 °C, 1 min at 60 °C, and 1 min at 72 °C. The experiments were performed in triplicates. The relative amount of cDNA copies was calculated using the modified Pfaffl model (Pfaffl 2001) ($Q = E^{{\Delta C_{t} }}$, where *E* is reaction efficiency and Δ*C*_*t*_ = *C*_*t* calibrator_ – *C*_*t* sample_). The calibrator sample was a mixture of several samples of total RNA of known concentration. The gene expression was normalized to the expression of three genes: *ATP6V1, HADHA,* and *UBE2D2,* selected by GeNorm program (ver. 3.5). After quality assessment, all data samples were used for final analysis.

### Singular value decomposition (SVD)

SVD is a standard method of linear algebra that may be used for revealing the major sources of variability in analyzed microarray dataset. By decomposition of data matrix into singular values (“patterns”), it allows to group the genes based on their gene expression profiles. As a result, small sets of original genes (modes) are selected and then hierarchical clustering of genes and samples for each gene modes is applied and presented on heat map plot (Simek and Kimmel 2003). The microarray analyses were performed using R environment (ver. 3.02) with the Bioconductor packages and MATLAB environment (ver. R2009B).

SVD was initially performed on the whole dataset, then using only serous and undifferentiated tumors. We decided to focus on the genes from the first mode of SVD done on serous and undifferentiated tumors. However, this set of ovarian cancers contained two series of surgical samples collected in different periods of time: 32 samples were collected in mid-1990s and 40 samples were collected in early 2000s . To avoid artifacts resulting from data heterogeneity, we did SVD in each series separately and choose only the transcripts that were common in both analyses (151 probe sets).

### Gene set enrichment analysis

Biological significance of all genes connected with two clusters with distinct survival (Fig. 3.) was performed using gene set enrichment analysis (GSEA) (Subramanian et al. 2005) with c2: curated gene set collections from Molecular Signatures Database (MSigDB) (Liberzon et al. 2011). In detail, we applied two independent tests: the LS permutation test and the Efron–Tibshirani gene set analysis test (GSA). We considered a GSEA category significantly differentially regulated if significance level in either of the tests was less than 0.05 after Benjamini–Hochberg false discovery rate (FDR) multiple test correction. The intersection of the GSA test and the LS permutation test was used. Analyses were performed using R (ver. 3.0.2) statistical environment with the Bioconductor software (ver. 2.13) and BRB-ArrayTools (developed by Dr. Richard Simon and the BRB-ArrayTools Development Team; ver. 4.4.0).

### Overall survival (OS) and disease-free survival (DFS) analyses

OS and DFS analyses were performed by the Kaplan–Meier method and compared between groups using the log-rank test. Differences in characteristics between groups of patients, according to the clusters obtained in microarray analysis and to quantitative PCR estimated gene expression levels, were evaluated by the χ2 test. A *p* value of <0.05 was considered statistically significant. The quantitative PCR validation was performed using the learning set and the test set samples. In the learning set, we have used the same samples as in the microarray experiment, and in the test set, we have used an independent set of 33 ovarian cancer samples. The analyses of survival time were performed using R Statistical Software.

## Results

### Histological tumor type is the major factor influencing gene expression profiles in ovarian cancer

**Fig. 1:**
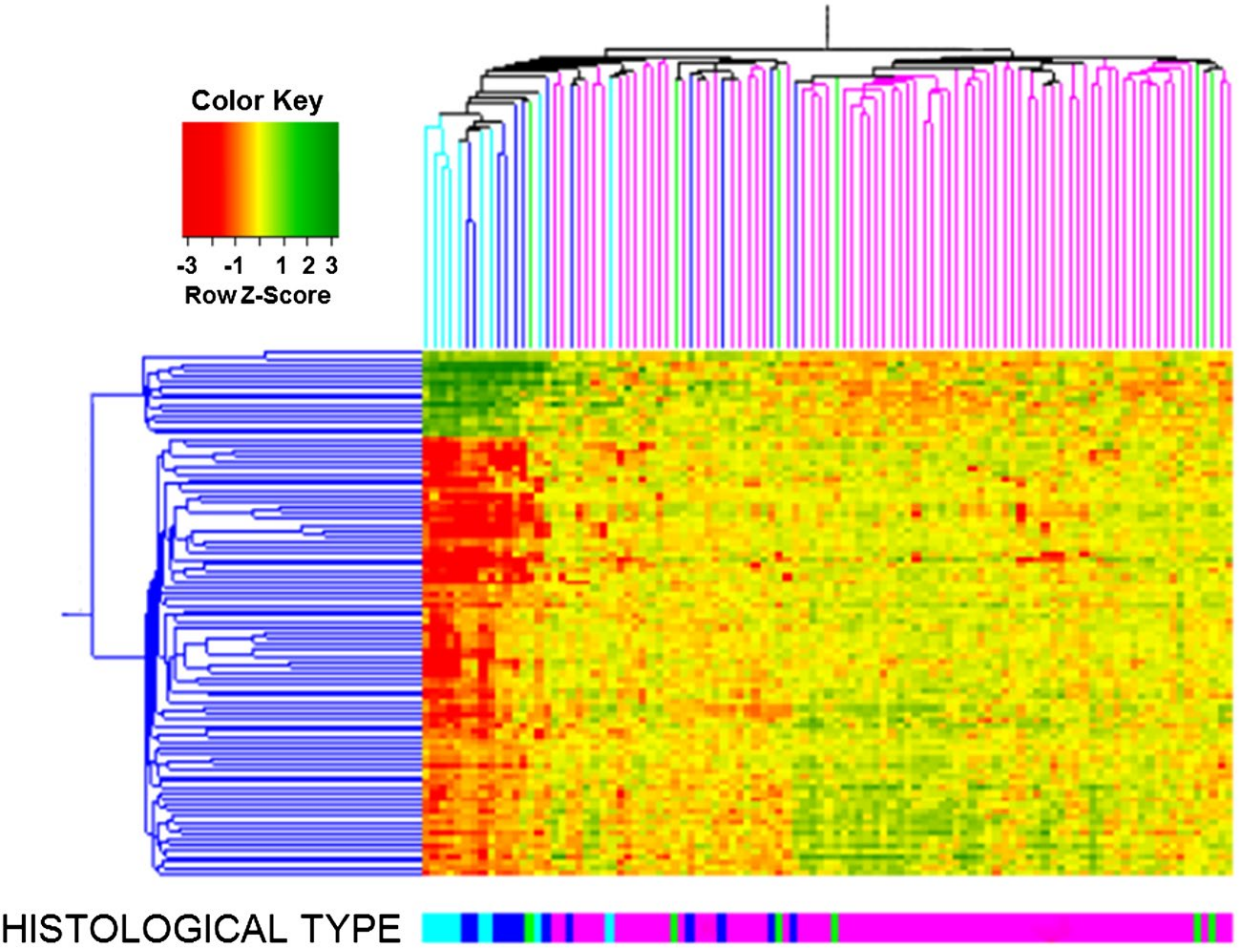
Hierarchical clustering of samples based on transcript expression levels from the first SVD mode. The SVD was done on all 101 cancer samples: 74 serous (*pink*), 12 endometrioid (*dark blue*), 9 clear cell (*light blue*), and 6 undifferentiated (*green*). Clear-cell and endometrioid cancers grouped together and showed common gene expression patterns that were distinct from those of the remaining tumor samples. Undifferentiated cancers were dispersed mostly among and had gene expression patterns similar to neighboring serous samples

We analyzed global gene expression in 101 ovarian cancer samples with an Affymetrix DNA microarray. The major intrinsic sources of variability in gene expression profiles were identified by SVD. The first SVD mode contained 92 probe sets, corresponding to 69 genes (Supplementary Table 2). A gene ontology analysis using GOHyperG Bioconductor Package revealed that the corresponding transcripts were primarily associated with cellular metabolism and proliferation along with signaling pathways that are implicated in development and reproduction. When we performed hierarchical clustering of the samples based on transcript expression levels, we observed that the clustering pattern was related to the histological type of tumor (Fig. 1). The left branch of the dendrogram contained all clear-cell tumors and all but one endometrioid tumors, as well as 23 serous tumors. The majority of clear-cell and endometrioid tumors were clustered together and showed common gene expression patterns that were distinct from those of other tumor samples. This was consistent with observations made in another microarray study (Marquez et al. 2005).

The right branch of the dendrogram contained mostly serous tumors (51 samples) and only one endometrioid tumor. Undifferentiated tumors were present in both branches; all but one were dispersed among and showed similar molecular profiles to neighboring serous tumors. The similarity in gene expression profiles between serous and undifferentiated cancers was also seen previously when supervised methods were applied (Lisowska et al. 2014).

### Extracellular matrix and immunological response constitute a second major source of variability in ovarian cancer

A second SVD mode representing the next major source of variability in the molecular profiles of the analyzed samples consisted of 116 probe sets corresponding to 77 genes (Supplementary Table 3). These transcripts were mainly associated with ECM organization, cell motility, adhesion, and immunological response. The clustering based on expression levels of these probe sets did not reveal any discernible patterns (not shown).

**Fig. 2:**
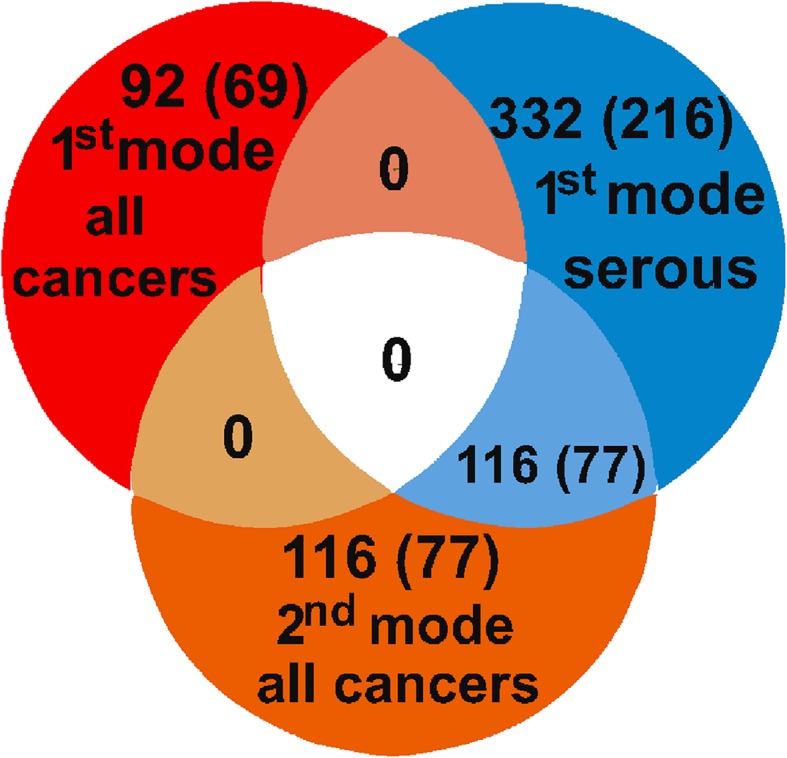
Relationship between SVD modes. *Venn diagram* shows the numbers of probe sets and genes (in *brackets*) obtained in SVD. All 116 probe sets in the second mode of SVD carried out on all tumors (*orange*) were among the 332 in the first mode of SVD, which was carried out on serous and undifferentiated tumors (*blue*). This suggests that the second mode of SVD done on all cancer samples, corresponded to the same biological feature(s) as the first mode of SVD done only on serous and undifferentiated cancers

Interestingly, when we repeated the SVD by taking into account only serous and undifferentiated tumors, the above-described gene signature re-emerged as the first SVD mode. In this setting, genes that were previously found in the second SVD mode now appeared in the first mode (Fig. 2).

After additional filtering of this gene signature (see Methods), we obtained 151 probe sets representing 111 unique sequences, among them 96 characterized genes (Table 3, Supplementary Table 4).

**Table 3:** List of characterized genes included in the 151-probe set signature

No.	Gene symbol	Gene description	Fold change
1	POSTN	**Periostin, osteoblast-specific factor**	**21.69**
2	COL11A1	**Collagen, type XI, alpha 1**	**19.44**
3	SFRP2	**Secreted frizzled-related protein 2**	**18.61**
4	DSPG3	**Dermatan sulfate proteoglycan 3/EPYC**	**13.17**
5	COL10A1	Collagen, type X, alpha 1 (Schmid metaphyseal chondrodysplasia)	10.24
6	ITGBL1	**Integrin, beta-like 1 (with EGF-like repeat domains)**	**8.1**
7	LOX	**Lysyl oxidase**	**7**
8	HNT	Neurotrimin	6.79
9	MFAP5	**Microfibrillar-associated protein 5**	**6.36**
10	FAP	**Fibroblast activation protein, alpha**	**6.22**
11	THBS2	Thrombospondin 2	6.02
12	COMP	Cartilage oligomeric matrix protein	5.18
13	CSPG2	Chondroitin sulfate proteoglycan 2 (versican, VCAN)	5.02
14	ASPN	Asporin (LRR class 1)	4.61
15	INHBA	**Inhibin, beta A (activin A, activin AB alpha polypeptide)**	**4.56**
16	CXCL14	Chemokine (C-X-C motif) ligand 14	4.5
17	LUM	Lumican	4.45
18	SULF1	Sulfatase 1	4.4
19	GJB2	Gap junction protein, beta 2, 26 kDa (connexin 26)	4.39
20	VCAM1	Vascular cell adhesion molecule 1	4.22
21	CTSK	Cathepsin K (pycnodysostosis)	4.18
22	MMP11	Matrix metallopeptidase 11 (stromelysin 3)	4.17
23	PRRX1	Paired related homeobox 1	4.08
24	TIMP3	TIMP metallopeptidase inhibitor 3 (Sorsby fundus dystrophy, pseudoinflammatory)	4.02
25	COL8A1	Collagen, type VIII, alpha 1	4.01
26	CXCL12	Chemokine (C-X-C motif) ligand 12 (stromal cell-derived factor 1)	3.95
27	SFRP4	Secreted frizzled-related protein 4	3.95
28	SERPINF1	Serpin peptidase inhibitor, clade F, member 1	3.88
29	TMEM158	Transmembrane protein 158	3.87
30	COL12A1	Collagen, type XII, alpha 1	3.85
31	COL5A2	Collagen, type V, alpha 2	3.85
32	FN1	Fibronectin 1	3.84
33	CCDC80	Coiled-coil domain containing 80	3.7
34	MEGF10	Multiple EGF-like domains 10	3.61
35	COL5A1	Collagen, type V, alpha 1	3.45
36	ADAM12	ADAM metallopeptidase domain 12 (meltrin alpha)	3.44
37	PLAU	**Plasminogen activator, urokinase**	**3.38**
38	EDIL3	EGF-like repeats and discoidin I-like domains 3	3.28
39	EDNRA	Endothelin receptor type A	3.26
40	LRRC15	Leucine-rich repeat containing 15	3.23
41	VGLL3	Vestigial like 3 (Drosophila)	3.21
42	AEBP1	AE binding protein 1	3.19
43	GLT8D2	Glycosyltransferase 8 domain containing 2	3.02
44	COLEC12	Collectin subfamily member 12	2.92
45	OLFML2B	Olfactomedin-like 2B	2.88
46	CRISPLD2	Cysteine-rich secretory protein LCCL domain containing 2	2.88
47	COL1A1	Collagen, type I, alpha 1	2.85
48	TNFAIP6	Tumor necrosis factor, alpha-induced protein 6	2.83
49	CTHRC1	Collagen triple helix repeat containing 1	2.74
50	EVI2A	Ecotropic viral integration site 2A	2.73
51	SNAI2	Snail homolog 2 (Drosophila)	2.72
52	FBN1	Fibrillin 1	2.69
53	PDLIM3	PDZ and LIM domain 3	2.69
54	GUCY1A3	Guanylate cyclase 1, soluble, alpha 3	2.67
55	DCN	Decorin/DSPG2	2.65
56	MMP2	Matrix metallopeptidase 2 (gelatinase A, 72 kDa type IV collagenase)	2.64
57	ALDH1A3	Aldehyde dehydrogenase 1 family, member A3	2.61
58	FYB	FYN binding protein (FYB-120/130)	2.57
59	COL3A1	Collagen, type III, alpha 1 (Ehlers–Danlos syndrome type IV)	2.55
60	SDC1	Syndecan 1	2.54
61	HSD17B6	Hydroxysteroid (17-beta) dehydrogenase 6	2.54
62	CDH11	Cadherin 11, type 2, OB-cadherin (osteoblast)	2.51
63	FNDC1	Fibronectin type III domain containing 1	2.43
64	MOXD1	Monooxygenase, DBH-like 1	2.43
65	PLN	Phospholamban	2.41
66	ISLR	Immunoglobulin superfamily containing leucine-rich repeat	2.37
67	TWIST1	Twist homolog 1 (Drosophila)	2.37
68	SPARC	Secreted protein, acidic, cysteine-rich (osteonectin)	2.37
69	ZFHX1B	Zinc finger homeobox 1b	2.35
70	THBS1	Thrombospondin 1	2.33
71	LR8	LR8 protein	2.33
72	MSRB3	Methionine sulfoxide reductase B3	2.32
73	FRMD6	FERM domain containing 6	2.23
74	TMEM46	Transmembrane protein 46	2.21
75	LY96	Lymphocyte antigen 96	2.19
76	HOXA3	Homeobox A3	2.18
77	WASPIP	Wiskott–Aldrich syndrome protein interacting protein	2.16
78	MICAL2	Microtubule associated monooxygenase, calponin and LIM domain containing 2	2.15
79	SAMSN1	SAM domain, SH3 domain and nuclear localization signals, 1	2.15
80	RAB31	RAB31, member RAS oncogene family	2.13
81	CALD1	Caldesmon 1	2.11
82	DEPDC7	DEP domain containing 7	2.09
83	BGN///TSHZ1	Biglycan///teashirt family zinc finger 1	2.06
84	LOXL2	Lysyl oxidase-like 2/ENTPD4	2.02
85	F2R	Coagulation factor II (thrombin) receptor	1.98
86	TFEC	Transcription factor EC	1.97
87	SLAMF8	SLAM family member 8	1.96
88	CYP7B1	Cytochrome P450, family 7, subfamily B, polypeptide 1	1.96
89	LAMA4	Laminin, alpha 4	1.96
90	COL6A3	Collagen, type VI, alpha 3	1.94
91	OLFML1	Olfactomedin-like 1	1.93
92	PLXNC1	Plexin C1	1.89
93	QKI	Quaking homolog, KH domain RNA binding (mouse)	1.85
94	NEXN	Nexilin (F actin binding protein)	1.82
95	SMOC2	SPARC related modular calcium binding 2	1.82
96	HEPH	Hephaestin	1.71

Genes are sorted by fold change; 10 genes selected for quantitative PCR validation are marked in bold

We investigated the cellular and molecular processes that may be affected by the differential expression of these 151 transcripts. Gene set enrichment analysis was performed based on MSigDB content (Supplementary Table 5). Among significantly affected signaling pathways we found, e.g., Biocarta: Fibrinolysis_Pathway, LYM_Pathway, CTL_Pathway and TCRA_Pathway; KEGG: ECM_Receptor_Interaction, Ribosome, and Focal_Adhesion; Reactome: Chondroitin_Sulfate_Biosynthesis, Collagen_Formation, Glycosaminoglycan_Metabolism, ECM_Organization, Degradation_of_ECM, Metabolism_of_Proteins, Translation, and Peptide_Chain_Elongation. There were also multiple curated gene sets overrepresented, which were found by other researchers to be related with cancer biology and tumor response to the therapy, e.g., Alonso_Metastasis_EMT_Up, Anastassiou_Cancer_Mesenchymal_Transition_Signature, Charafe_Breast_Cancer_Basal_vs_Mesenchymal_Down, Cowling_MYCN_Targets, Croonquist_NRAS_vs_Stromal_Stimulation_Down, Dasu_IL6_Signalling_Down, Hernandez_Mitotic_Arrest_by_Docetaxel, Mahajan_Response_to_IL1A_Down, Mishra_Carcinoma_Associated_Fibroblast_Up, Nakamura_Cancer_Microenvironment_Up, Pid_AVB3_Integrin_Pathway, etc.

### Two clusters of ovarian cancers with distinct survival

**Fig. 3:**
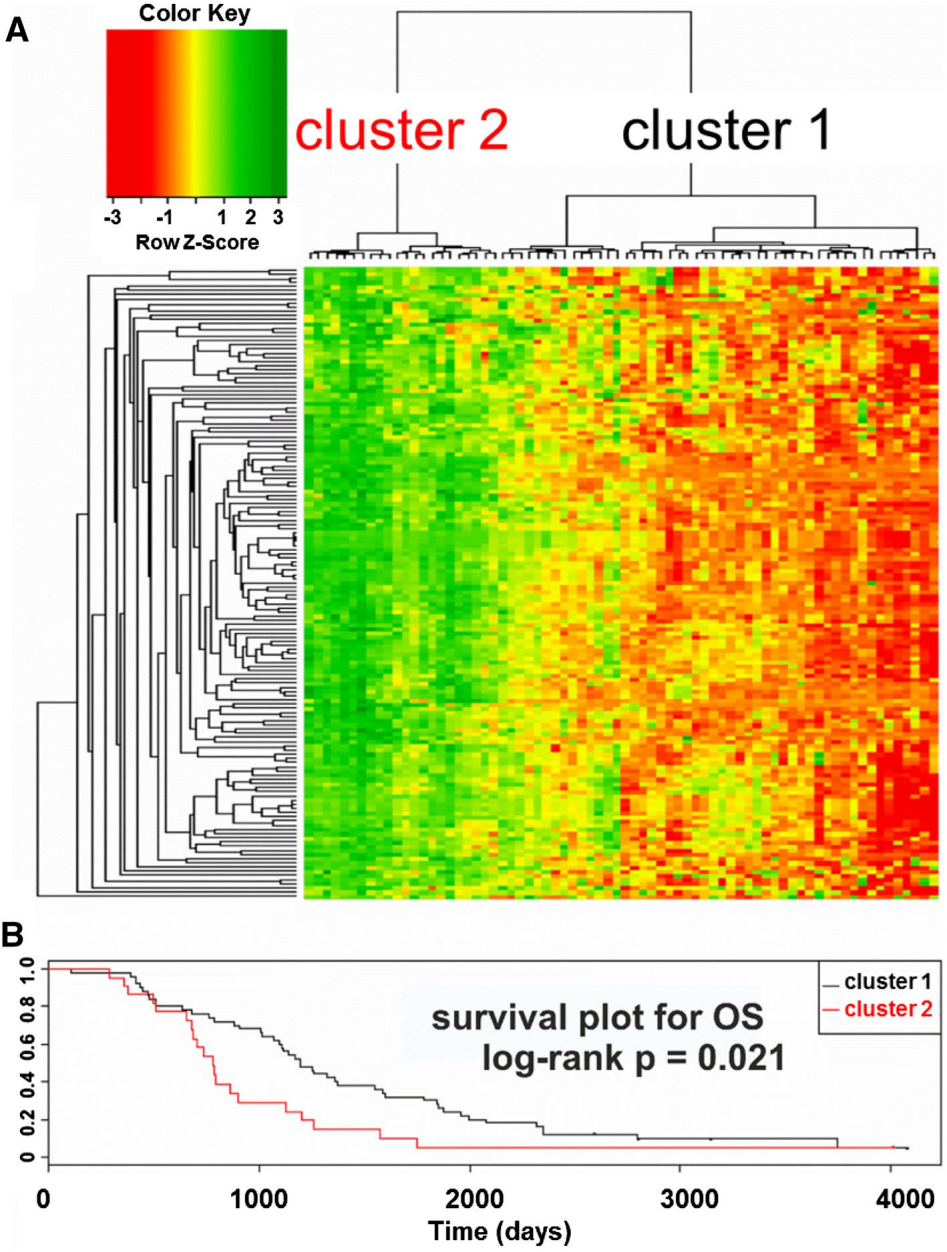
**a** Hierarchical clustering based on the expression of the 151-probe set signature revealed two clusters of ovarian cancer with distinct molecular profiles. Four undifferentiated and 68 serous samples with complete clinical and molecular data were used for clustering. **b** The Kaplan–Meier survival analysis of patient OS was carried out using the log-rank test for each cluster. The two clusters were characterized by different OS (*p* = 0.021). Patients who had tumors with higher expression of the 151 transcripts (cluster 2) had shorter OS [median value = 735, 1 quartile range (QR) = 652, 3 QR = 897], while those with tumors showing lower expression of these genes (cluster 1) had longer OS (median value = 1194.5, 1 QR = 767.25, 3 QR = 1867.75)

Hierarchical clustering based on the expression of the aforementioned 151 transcripts revealed two unequal clusters of ovarian cancer samples (defined by two major sub-branches of dendrogram), with strikingly different molecular profile (Fig. 3a). Cluster 1 (right sub-branch of dendrogram) was larger (50 samples) and characterized by lower expression values of those genes. Cluster 2 (left sub-branch) was smaller (22 samples) and showed higher expression values. We found that samples representing those two clusters did not differ with any of the following features: tumor stage, tumor grade, response to chemotherapy, residual tumor size, germline *breast cancer* (*BRCA*)*1* mutation, somatic *p53* mutation, or p53 protein accumulation. However, the Kaplan–Meier analysis revealed that patients from the two clusters exhibited statistically significant difference in OS (Fig. 3b). For DFS, we observed similar trend, although it was not statistically significant (not shown).

### Factors involved in clustering pattern and difference in survival

**Fig. 4:**
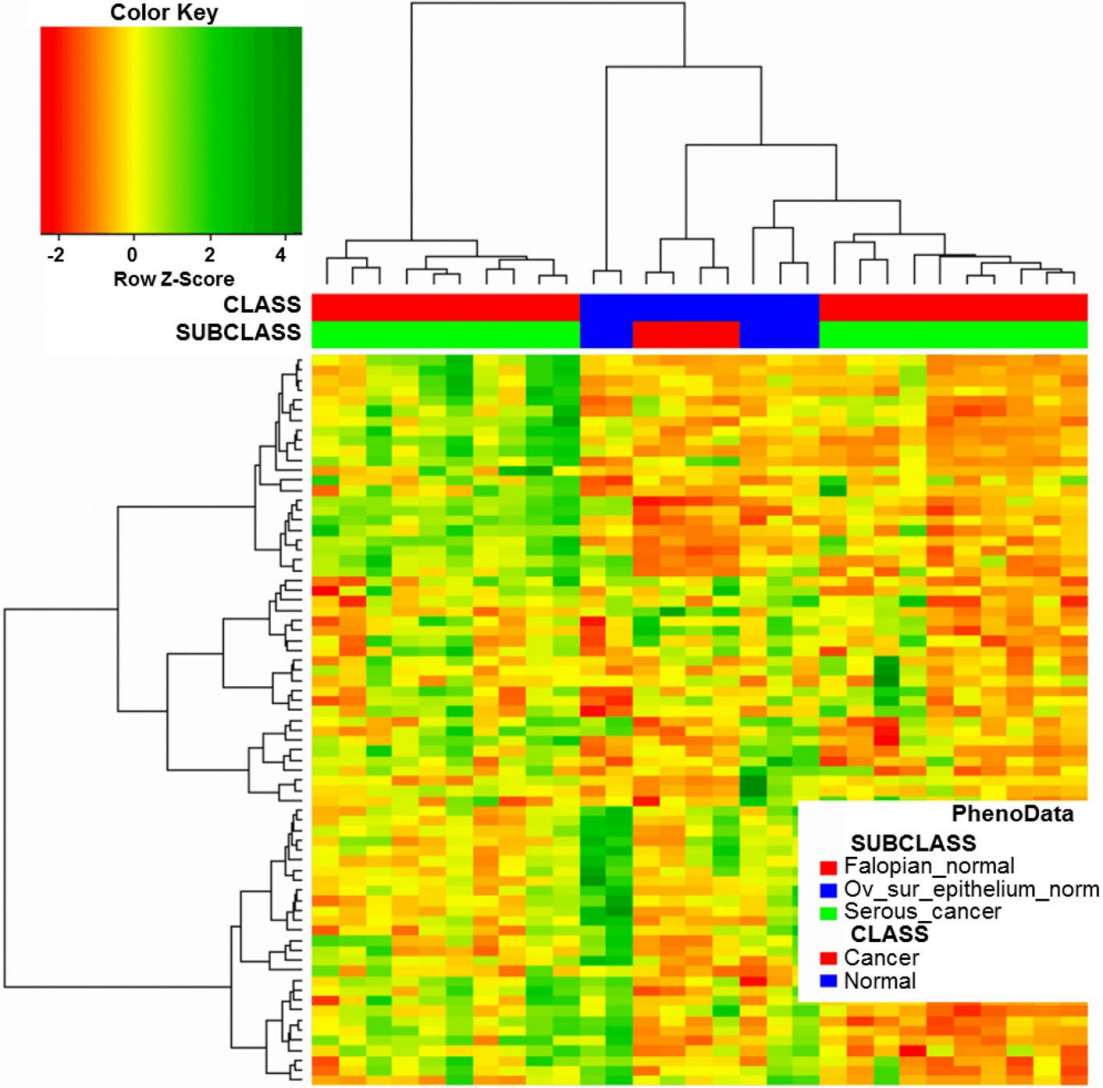
Hierarchical clustering of cancer and normal samples from (Marquez et al. 2005) based on the expression levels of our 151-probe set signature [only 73 probe sets matched due to the older version of the array used in (Marquez et al. 2005)]. Serous ovarian cancers from Marquez study were divided into two clusters; however, normal controls were not, and there was no relationship between the expression patterns of either cluster and particular type of normal control

We investigated whether the 151-probe set signature and corresponding clustering pattern were due to the potentially different cellular origin of ovarian cancers (i.e., ovarian or fallopian epithelial). We used previously reported microarray data that included different histological types of ovarian cancer as well as normal ovarian and normal tubal epithelial samples (Marquez et al. 2005). We used our 151-probe set signature for hierarchical clustering of 20 serous cancers, five ovarian surface epithelial samples, and 4 fallopian tube epithelial samples from the Marquez study. We predicted that if our signature detects differences between serous ovarian cancers originating from distinct epithelia, the clustering pattern would reveal the relationship between them and corresponding normal epithelium. However, we did not observe any such pattern (Fig. 4).

**Fig. 5:**
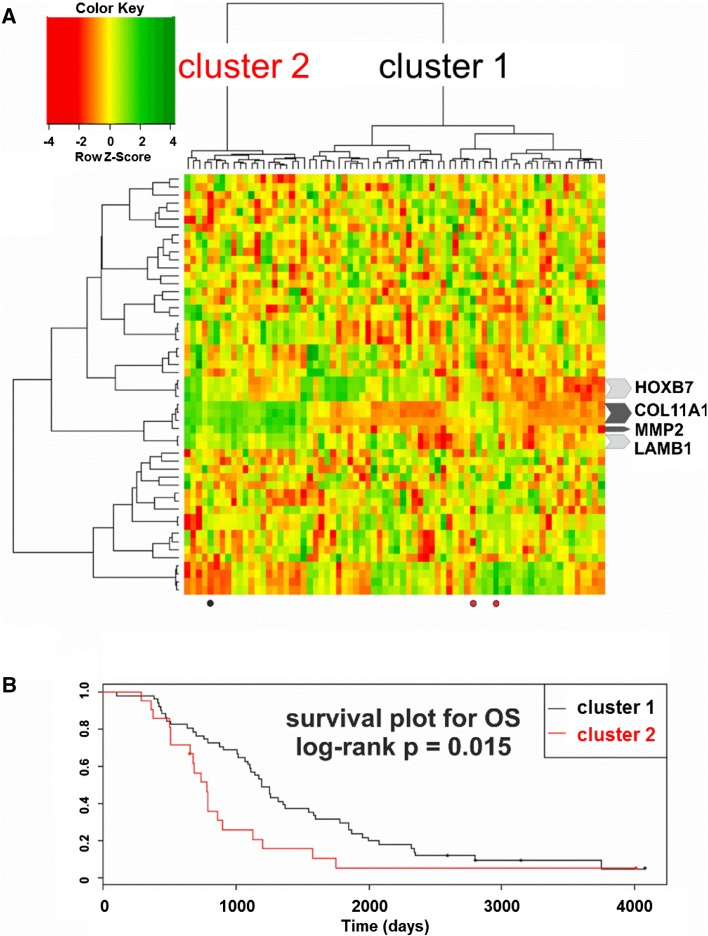
**a** Hierarchical clustering of serous and undifferentiated cancer samples from our experiment using a previously reported gene signature for the malignant potential of ovarian tumors (Ouellet et al. 2005). The clustering pattern was very similar to that obtained using our 151-probe set signature owing to the expression patterns of the only two genes common to the two signatures (*COL11A1* and *MMP2*). Similar expression patterns were observed for *laminin beta 1* and *homeobox B7*, but other genes showed random patterns. Dots indicate tumor samples that were clustered in a different manner from the analysis carried out using our signature: *red* and *black dots* indicate samples that were previously included in clusters 2 and 1, respectively. **b** Kaplan–Meier survival analysis of patient OS (log-rank test) based on cluster (*P* = 0.015)

We also assessed whether the observed clustering patterns and differences in survival were related to the malignant potential of tumors. We applied to our data a previously reported gene signature (Ouellet et al. 2005) that distinguished between low malignant potential versus invasive epithelial tumors. Interestingly, we obtained an almost identical clustering pattern as when we used our 151-probe set signature, with patient OS differing significantly between the two clusters (Fig. 5); this pattern contained 21 and 51 samples, only three of which were clustered differently from what was observed using our signature. The obtained clustering pattern was primarily based on the expression of three probe sets for *collagen type XI alpha* (*COL11A*)*1* and one for *matrix metalloproteinase* (*MMP*)*2*. Notably, these were the only genes that were common to the Ouellet signature and ours. In addition, only these four probe sets behaved consistently in relation to our expression data, showing low and high expression in clusters 1 and 2, respectively.

### Candidate prognostic markers

We analyzed patients with serous and undifferentiated cancers based on standard clinical prognostic factors (tumor grade, disease stage, and residual tumor size) and found that prognosis was similar for whole group. However, molecular profiles delineated two subgroups with different OS (Fig. 3). Patients with shorter survival had tumors with higher expression of the 151 probe sets, while those with longer survival had tumors with lower expression, suggesting that corresponding genes are potential prognostic markers.

We examined 10 genes from the 151-probe set signature in terms of their ability to predict patient OS. Genes were selected arbitrarily, considering two factors: significant differences in expression level between clusters (fold change, FC) and/or established/suggested role in cancer. The majority of selected genes met the criterion of FC > 5, with only *inhibin beta A* (*INHBA*) and *plasminogenactivator urokinase* (*PLAU*) showing lower FC values (Table 4).

**Table 4:** Results of quantitative PCR validation of selected genes

Gene symbol	Gene name	Overall survival (*p* value)		Disease-free survival (*p* value)	
		Learning set^a^	Test set^b^	Learning set	Test set
*POSTN*	Periostin	0.3933	**0.0077**	0.7152	**0.0259**
*COL11A1*	Collagen type XI alpha 1	**0.0020**	0.1355	*0.0647*	0.2254
*SFRP2*	Secreted frizzled-related protein 2	0.8918	*0.0715*	0.6884	*0.0574*
*DSPG3*	Dermatan sulfate proteoglycan 3	**0.0032**	**0.0140**	**0.0328**	**0.0053**
*ITGBL1*	Integrin beta-like 1	0.2123	0.2989	0.9827	0.7200
*LOX*	Lysine oxidase	**0.0099**	**0.0363**	*0.0592*	**0.0485**
*MFAP5*	Microfibrillar-associated protein 5	**0.0016**	0.1246	*0.0671*	*0.0974*
*FAP*	Fibroblast activating protein	**0.0025**	0.1921	0.2637	0.7907
*INHBA*	Inhibin beta A	0.1581	*0.0855*	0.2797	0.2435
*PLAU*	Plasminogen activator urokinase	0.2092	**0.0347**	0.4369	0.1347

*p* < 0.05 is indicated in bold; *p* < 0.10 is indicated in italics. Survival analyses in the learning set were carried out in relation to the threshold expression between the two cancer sample clusters. In the test set, survival was analyzed in relation to median expression

^a^Set of samples used for microarray experiment (*n* = 72)

^b^Set of samples from the independent group of ovarian cancer patients (*n* = 33)

We first performed quantitative PCR measurement of genes expressed in the RNA samples that were analyzed by microarray (learning set). Five genes were positively validated with respect to OS: *lysine oxidase* (*LOX*), *microfibrillar*-*associated protein* (*MFAP*)*5*, *fibroblast activating protein (FAP*), *dermatan sulfate proteoglycan* (*DSPG*)*3*, and *COL11A1* (Table 4; Supplementary Fig. 1). We then verified 10 selected genes in the independent set of ovarian cancer samples (test set) and found *LOX* and *DSPG3* to be significant. In addition, *periostin* (*POSTN*) and *PLAU* were associated with OS in the test set while *secreted frizzled*-*related protein* (*SFRP*)*2*, *thrombospondin 2*, and *INHBA* were close to significance (Table 4; Supplementary Fig. 2).

We then analyzed gene expression with respect to DFS in the learning and test sets. In the former, *DSPG3* was significant, whereas *COL11A1*, *LOX*, and *MFAP5* showed similar trend and were close to significance (Table 4; Supplementary Fig. 3). *DSPG3* was also significant in the test set along with *LOX*, while *MFAP5* and *SFRP2* were close to significance (Table 4; Supplementary Fig. 4).

In summary, two genes—i.e., *DSPG3* and *LOX*—were significantly associated with OS and DFS in the learning and test sets of ovarian cancer samples. Several other genes showed trend toward significance.

## Discussion

Many microarray studies rely only on supervised analyses that compare predefined classes of samples. In this study, we used singular value decomposition, an unsupervised method of data analysis that does not need predefining any classes. It identifies, by itself, the strongest, intrinsic sources of variability in the analyzed dataset, which can be then examined in relation to clinicopathological features and biological significance. In addition, SVD technique allows detection and elimination of unwanted “noise” in the microarray data resulting from technical variability or from other undefined sources of heterogeneity. This approach allowed successful characterization of the analyzed set of ovarian cancers and identification of several potential prognostic biomarkers.

### Histological type of tumor influences gene expression in ovarian cancer

When we applied SVD to samples comprising different histological types of ovarian cancer, we observed that the first SVD mode—which represents the greatest source of variability in gene expression patterns—was associated with histological type. These results are in accordance with our previous supervised analyses, which showed that the histological type of a tumor was the factor which caused the greatest change in gene expression (3526 differentially expressed probe sets; FDR < 10 %) (Lisowska et al. 2014). In contrast, in breast cancer, we found only 11 probe sets that were differentially expressed between two histological types (ductal and medullary; FDR < 10 %) (Dudaladava et al. 2006; Lisowska et al. 2011). Therefore, it seems that the histological type of a tumor is not a universal source of variability in gene expression patterns in cancer. In ovarian cancer, these differences may be enhanced by the distinct cellular origin of histological tumor types; a growing body of evidence suggests that clear-cell and endometrioid cancers develop from endometriosis, while serous and undifferentiated tumors originate from tubal or ovarian epithelium (Chan et al. 2012; Erickson et al. 2013; Jones and Drapkin 2013; Kurman and Shih Ie 2011).

Our results also lead to some practical conclusions. We observed that there were many genes shared between clear-cell and endometrioid but not serous cancer (Lisowska et al. 2014). On the other hand, serous and undifferentiated tumors had near-identical gene expression profiles, as confirmed by SVD. Therefore, based on their molecular similarity, we merged serous and undifferentiated tumors into a single group, whereas clear-cell and endometrioid cancers—representing molecular entities distinct from the two former types of tumor—were excluded from further analyses.

Large differences in gene expression profiles between various histological types of ovarian cancer have already been noted in other microarray studies, but to our knowledge, they have never been regarded as a confounding factor when analyzing other features. Moreover, in many studies, a search for molecular mechanisms underlying tumor features such as chemoresistance has been carried out across different histological types (Helleman et al. 2006; Jazaeri et al. 2005). We presume that such studies would produce more reliable results if carried out on a histologically homogeneous group of samples.

### The 151-probe set signature overlaps with an invasion-associated signature related to stromal desmoplastic reaction

The second major source of variability identified by SVD was associated with the expression of a set of genes related to the ECM, cell motility, adhesion, and immunological response. This signature emerged as a second SVD mode when all histological types of tumor were analyzed, and became a dominant hallmark when only serous/undifferentiated tumors were taken into account. Interestingly, we found considerable match of this gene signature with a gene set described in the study (Kim et al. 2010), which analyzed several tumor expression datasets with clinical staging information, available in the public databases, among them ovarian dataset (Bignotti et al. 2007). Described gene set was co-expressed with *COL11A1* and was reportedly observed in different types of cancer (ovarian, colon, breast, pancreatic, and gastric).

In our 151-probe set signature, 68 probe sets (representing 42 genes) were found to overlap with a previously reported 100-probe set signature, i.e., “Aggregate list of top genes associated with *COL11A1*” (Kim et al. 2010) (Supplementary Table 4); 68 % of these probe sets were present in our signature. These authors postulated that this signature was a hallmark of invasion-associated desmoplastic reaction, which is acquired by various cancers at a different clinical stages (e.g., at stage IIIC in ovarian and stage II in colorectal cancer). Indeed, we observed a greater proportion of highly advanced stages within cluster 2, which had shorter survival; however, this difference was not significant (Table 2).

Several genes from this signature were validated by quantitative PCR, suggesting that they can be potentially useful as prognostic markers. The slight discrepancy in the validation results between the two sets of samples may be due to the small size of the independent set. Second reason may be connected with different median survival times of the patients from learning set (earlier cohort of patients: some treated with platinum/cyclophosphamide, some with taxane/platinum regimen, TP) and from the test set (patients uniformly treated with TP) (Supplementary Fig. 5).

### The two identified clusters are unrelated to the cellular origins of ovarian cancer

Serous ovarian cancers are increasingly viewed as having mixed epithelial etiology (ovarian or tubal) (Erickson et al. 2013). We therefore assessed whether the two clusters of cancer with distinct OS identified in our study were of different cellular origins. Only one study to date has investigated the gene signature of normal cells of origin in ovarian cancer (Merritt et al. 2013). A comparison of gene expression profiles between normal fallopian and normal ovarian epithelia revealed 632 probe sets overexpressed in the former and 525 overexpressed in the latter; patients who had tumors with a fallopian signature had significantly shorter OS and DFS than those with an ovarian signature. However, we found only one fallopian signature gene in our 151 probe sets. We also examined, using previously published microarray data (Marquez et al. 2005), whether our 151-gene probe set signature can discriminate between ovarian and fallopian epithelial samples and identify fallopian-like and ovarian-like cancers. Obtained clustering results (Fig. 4) supported the view that our prognostic signature is unrelated to the cellular origin of ovarian cancer. Interestingly, serous cancers from Marquez study formed two clusters based on the expression of genes from our prognostic signature; however, we were unable to verify whether these clusters are related to OS due to the lack of survival data.

### Relationship between the two clusters and high- versus low-grade difference

Low- versus high-grade difference, also referred to as type I versus type II tumor difference (Vang et al. 2009), is a reliable prognostic factor for serous ovarian cancer. It is generally accepted that low-grade serous ovarian carcinomas (LG-SOC) develop from benign precursors, grow slowly, are genetically stable, and have good prognosis. In contrast, high-grade serous ovarian carcinomas (HG-SOC) and undifferentiated carcinomas—which are characterized by *p53* and *BRCA1/2* mutations and genomic instability—present at an advanced stage, evolve aggressively, and have poor prognosis.

We analyzed whether the two clusters of cancers with different OS that were observed in our study may be related to the difference between HG- and LG-SOC. In general, high-grade tumors were prevalent in the set of cancers used for hierarchical clustering (Table 2). Cluster 2, which is associated with shorter OS, contained more high-grade cancers than cluster 1, although this difference was not significant. Both clusters had similar numbers of *p53*-mutated tumors. Unexpectedly, there were more *BRCA1* mutations in cluster 1—which is associated with longer survival—than in cluster 2. This may result from the fact that tumors with BRCA1 mutation have impaired DNA repair, improved response to platinum compounds and thus better survival (Long and Kauff 2011). Taken together, these findings suggest that our prognostic signature is unrelated to HG- versus LG-SOC difference.

### The 151-probe set signature is presumed to be expressed by cancer cells and to confer chemoresistance

The *COL11A1*-related signature may be attributed to the presence of CAFs within the tumor (Kim et al. 2010). However, given that we made every effort to reduce the stromal component to below 15 %, the differential expression of the 151-gene probe sets is not likely caused by variable CAF content in our samples. We also found by semiquantitative reverse transcription PCR (RT-PCR) that 13 genes from Table 3 were expressed in at least two of the six established ovarian cancer cell lines that were analyzed (Supplementary Fig. 6). We therefore presume that neither the *COL11A1* signature (Kim et al. 2010) nor our 151-gene probe set prognostic signature is solely attributable to CAFs, but may in fact be expressed by cancer cells.

Three recent in vitro studies (Cheon et al. 2014; Januchowski et al. 2014; Wu et al. 2015) also provide evidence that similar gene sets (collagen/stromal related) may be expressed by cancer cells; moreover, two of these investigations suggest that these signatures are associated with ovarian cancer cell chemoresistance. A 10-gene collagen remodeling signature linked to poor outcome in serous ovarian cancer was induced by transforming growth factor-β1 in two ovarian cancer cell lines (OVCAR3 and A2780) (Cheon et al. 2014); nine of these genes overlapped with our 151-probe set signature. A comparison of gene expression profiles between wild-type and chemoresistant variants of W1 ovarian cancer cells identified a 10-gene signature overexpressed in the chemoresistant lines, with five of the genes overlapping with our signature (Januchowski et al. 2014). *COL11A1* was found to be upregulated in chemoresistant variants of OVCAR4 and IGROV1 cell lines relative to chemosensitive counterparts (Wu et al. 2015); 16 of the 30 genes overexpressed in the resistant cells were the same as those in our signature.

Two clinical studies have also implicated a similar stromal-related gene signature in ovarian cancer chemoresistance (Karlan et al. 2014; Ryner et al. 2015). One of these reports found that a *POSTN*-associated signature that included seven genes present also in our signature was linked to primary chemoresistance in ovarian cancer patients (Ryner et al. 2015); although these authors described *POSTN* expression only in the peritumoral stroma, we detected its expression by immunohistochemistry in a large subset of analyzed tumors (unpublished).

When we used a signature related to the malignant potential of ovarian tumors (Ouellet et al. 2005) to cluster our serous/undifferentiated cancer samples, we obtained a clustering pattern almost identical like with our 151-probe set signature that was entirely due to the expression patterns of, *COL11A1* and *MMP2*, the only two genes common to both signatures. Taken together, our findings suggest that *COL11A1* and co-expressed genes may play a significant role in the molecular evolution of ovarian tumors from low to highly aggressive, and in acquiring chemoresistance, which could explain the association between our 151-probe set signature and patient survival.

## Conclusions

We distinguished two clusters of serous ovarian cancers characterized by distinct OS using an unsupervised method of microarray data analysis. The two clusters did not derive from a high-grade versus low-grade difference in serous carcinomas, nor were they related to different histological origins of serous ovarian cancers (ovarian vs. fallopian). Our prognostic signature comprising 151 probe sets differentially expressed between the two clusters included mostly genes that were related to ECM structure and functions and immunological response; two of these—*DSPG3* and *LOX*—were validated by quantitative PCR in the initial and independent sets of ovarian cancer samples and were associated with OS and DFS. Interestingly, our prognostic signature showed considerable overlap with a recently described invasion-associated signature related to stromal desmoplastic reaction that emerged in advanced stages of different cancers and was linked to CAFs infiltration, although our tumor samples had a stromal component of <15 %. We also found that ovarian cancer cells from established lines express several genes from this signature. Therefore, we presume that this gene signature is attributable to ovarian cancer cells and may be related to their acquisition of chemoresistance, as suggested by other studies.

In comparison with our previous study, we demonstrated that unsupervised methods of microarray data analysis are more effective than supervised methods in identifying intrinsic, biologically sound sources of variability. Thus, it seems that they should be more widely applied in the molecular profiling of cancer. We also confirmed our previous observation that histological type of the tumor is the greatest source of variability in ovarian cancer and may interfere with analyses of other features. Thus, it is reasonable to use histologically homogeneous groups of ovarian cancer samples in microarray experiments.

## Electronic supplementary material

Below is the link to the electronic supplementary material.

Supplementary material 1 (PDF 234 kb)

Supplementary material 2 (PDF 222 kb)

Supplementary material 3 (PDF 220 kb)

Supplementary material 4 (PDF 222 kb)

Supplementary material 5 (PDF 90 kb)

Supplementary material 6 (PDF 251 kb)

Supplementary material 7 (PDF 17 kb)

Supplementary material 8 (PDF 44 kb)

Supplementary material 9 (PDF 46 kb)

Supplementary material 10 (XLSX 65 kb)

Supplementary material 11 (PDF 67 kb)
