# Supplementary material for: Unsupervised analysis reveals two molecular subgroups of serous ovarian cancer with distinct gene expression profiles and survival
Source: J Cancer Res Clin Oncol. 2016 Mar 30;142(6):1239–52. doi: 10.1007/s00432-016-2147-y (PMC4869753; doi:10.1007/s00432-016-2147-y)

Supplementary Figure 4.

Quantitative PCR validation of selected genes in relation to disease-free survival (DFS) in test set. The Kaplan-Meier plots of observed DFS for patients with ovarian cancer by log-rank test according to real-time RT-PCR estimated gene expression. Survival analyses in the test set were carried out in relation to median expression.

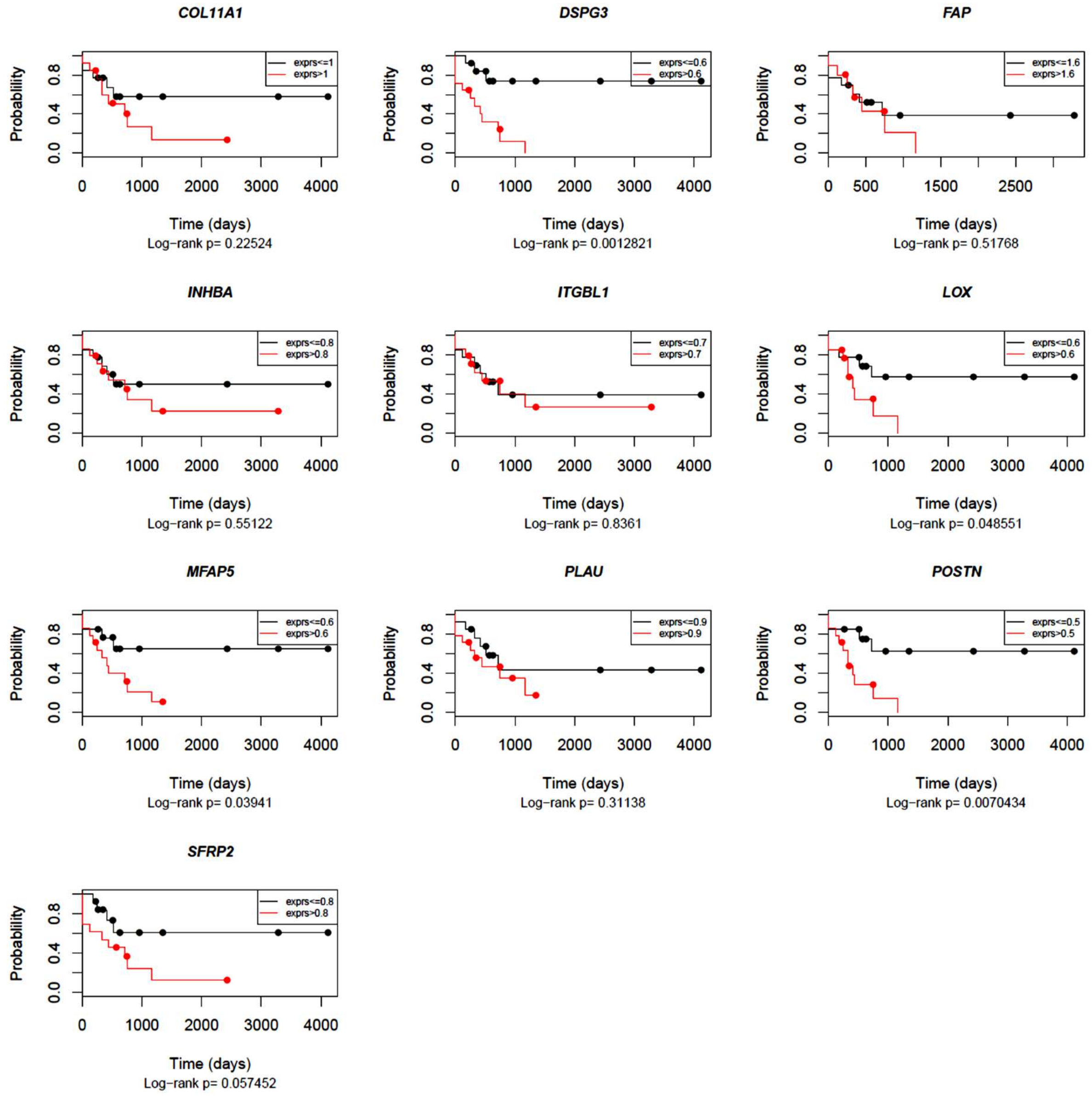

Supplement: Supplementary file 4 — Supplementary material 4 (PDF 222 kb) [file 432_2016_2147_MOESM4_ESM.pdf]
