# Supplementary material for: Unsupervised analysis reveals two molecular subgroups of serous ovarian cancer with distinct gene expression profiles and survival
Source: J Cancer Res Clin Oncol. 2016 Mar 30;142(6):1239–52. doi: 10.1007/s00432-016-2147-y (PMC4869753; doi:10.1007/s00432-016-2147-y)

Supplemetary Figure 5.

Different median OS and DFS of the patients from learning set and from test set.

Learning set consisted of the earlier cohort of patients: some were treated with platinum/cyclophosphamide, some with taxane/platinum regimen, TP), while the test set consisted of patients uniformly treated with TP and had better survival.

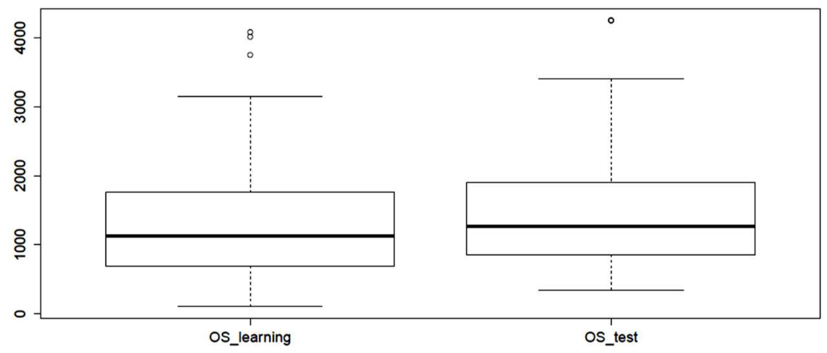

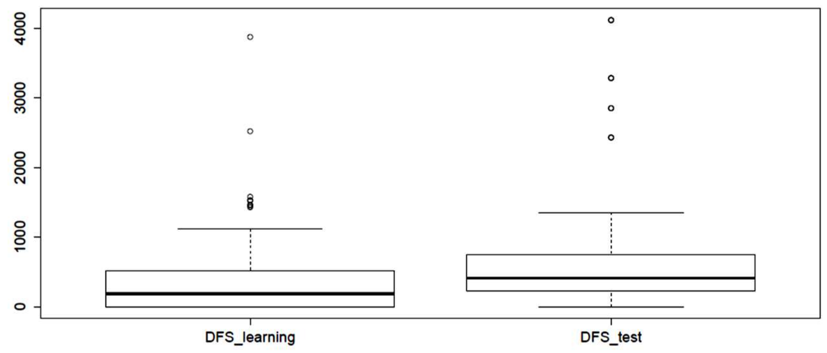

Supplement: Supplementary file 5 — Supplementary material 5 (PDF 90 kb) [file 432_2016_2147_MOESM5_ESM.pdf]
