# Supplementary material for: Unsupervised analysis reveals two molecular subgroups of serous ovarian cancer with distinct gene expression profiles and survival
Source: J Cancer Res Clin Oncol. 2016 Mar 30;142(6):1239–52. doi: 10.1007/s00432-016-2147-y (PMC4869753; doi:10.1007/s00432-016-2147-y)
Supplement: Supplementary file 6 — Supplementary material 6 (PDF 251 kb) [file 432_2016_2147_MOESM6_ESM.pdf]

Supplementary Figure 6.

Expression of selected genes from 151-probe set prognostic signature in 6 established ovarian cancer cell lines, as detected by semi-quantitative PCR. Five cell lines are commercially available (ATCC), while OVPA8 cell line has been established by us (derived from ascites of a patient with high grade serous ovarian carcinoma). The 18SrRNA was used as an internal control of RNA uniformity in the analyzed samples. Primers' sequence and RT-PCR details are available on request.

Lp.	Gene (amplicon)	Ovarian cancer cell line					
		A2780	ES2	OAW42	OVCAR3	OVPA8	SKOV3
1	POSTN (347 bp)	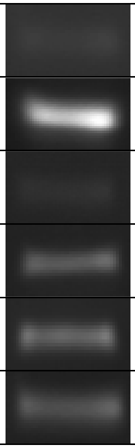	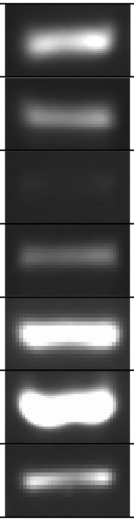	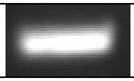	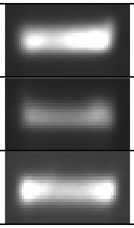	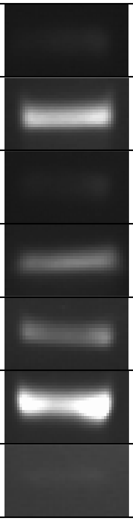	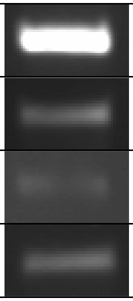
2	COL11A1 (396 bp)			lack of expression			
3	SFRP2 (300 bp)			lack of expression			
4	DSPG3 (368 bp)			lack of expression	lack of expression		
5	ITGBL1 (300 bp)			lack of lack of expression	lack of expression		lack of expression
6	LOX (460 bp)			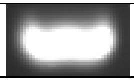	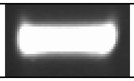		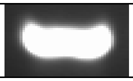
7	HNT (415 bp)	lack of expression		lack of expression	lack of expression		lack of expression
8	MFAP5 (452 bp)	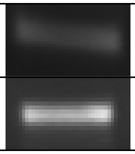	lack of expression	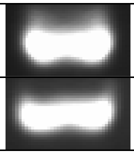	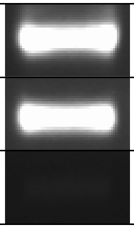	lack of expression	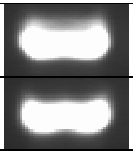
9	CSPG2/VCAN (473 bp)		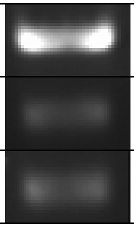			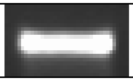	
10	FAP (366 bp)	lack of expression		lack of expression		lack of expression	lack of expression
11	THBS2 (339 bp)	lack of expression		lack of expression	lack of expression	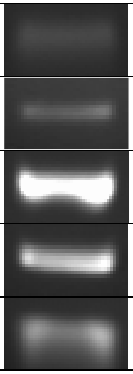	lack of expression
12	COMP (343 bp)	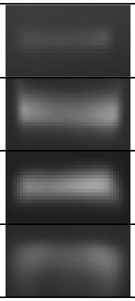	lack of expression	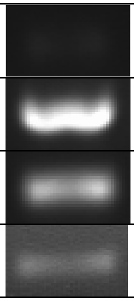	lack of expression		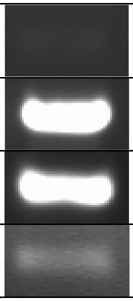
13	FN1 (493 bp)		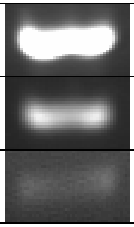		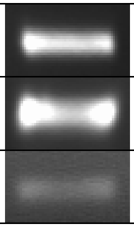		
14	PLAU (316 bp)						
15	18SrRNA (193 bp)						
