# Supplementary material for: Unsupervised analysis reveals two molecular subgroups of serous ovarian cancer with distinct gene expression profiles and survival
Source: J Cancer Res Clin Oncol. 2016 Mar 30;142(6):1239–52. doi: 10.1007/s00432-016-2147-y (PMC4869753; doi:10.1007/s00432-016-2147-y)
Supplement: Supplementary file 7 — Supplementary material 7 (PDF 17 kb) [file 432_2016_2147_MOESM7_ESM.pdf]

Supplementary Table 1.
The sequence of the primers and the numbers of Roche probes used for quantitative PCR

Gene	Probe no (Roche)	Forward primers (5' – 3')	Reverse primers (5' – 3')
**Experimental genes**			
COL11A1	#17	tcctcttccaagctagagaggtc	ggagaattgtgaaaatctagtgctt
COMP	#38	gcaccgacgtcaacgagt	tggtgttgatacagcggact
CSPG2	#54	gcacctgtgtgccaggata	cagggattagagtgacattcatca
DSPG3	#57	caggagcctgaattcacagg	acacaaaagacaggttggaaagt
FAP	#78	tttcaaatgtggtatagcagtgg	tttgttgggagacccatga
FN1	#52	gcgagagtgcccctactaca	gttggtgaatcgcaggtca
HNT	#32	tggagacacatctctcccaaa	ggaggcactgcactcgtag
INHBA	#22	tcacgtttgccgagtcag	gacttttaggaagagccagacttc
ITGBL1	#65	ttgctctgggaggggtaaa	ctcacaagtcttgccatacacc
LOX	#82	tgggaatggcacagttgtc	aaacttgctttgtggccttc
MFAP5	#21	gccaaatctgaagtcctttcc	cctactttggctggcgaata
PLAU	#46	ttgctcaccacaacgacatt	ggcaggcagatggtctgtat
POSTN	#20	tggtgtgatccatttgattga	cgtgaaggtggtttgctgt
SFRP2	#83	ctagcagcgaccacctcct	ttgcaggcttcacataccttt
THBS2	#17	gtgcaggagcgtcagatgt	gggttggataaacagccatc
**Reference genes**			
ATP6V1	#03	aagccggctggatctcat	gcatttgcaccaaacaagg
HADHA	#65	gtcttgcgcccatgatgt	cagcttcttcgggtcaactc
UBE2D2	#67	aatggcagcatttgtcttga	cacaacagagaacagatgga
