# Supplementary material for: Unsupervised analysis reveals two molecular subgroups of serous ovarian cancer with distinct gene expression profiles and survival
Source: J Cancer Res Clin Oncol. 2016 Mar 30;142(6):1239–52. doi: 10.1007/s00432-016-2147-y (PMC4869753; doi:10.1007/s00432-016-2147-y)
Supplement: Supplementary file 8 — Supplementary material 8 (PDF 44 kb) [file 432_2016_2147_MOESM8_ESM.pdf]

Supplementary Table 2.
First mode of SVD done on all tumors (101 ovarian cancer samples; 4 histological types: 74 serous, 12 endometrioid, 9 clear cell and 6 undifferentiated ovarian cancers). This SVD mode contained 92 probe sets (69 genes). Hierarchical clustering of cancer samples according to the expression of these genes revealed clustering pattern related with histological type of tumor.

No	Affymetrix probe set	Gene symbol	Gene name
1	1569555_at	GDA	guanine deaminase
2	241031_at	C2CD4A	family with sequence similarity 148, member A
3	235638_at	RASSF6	Ras association (RalGDS/AF-6) domain family member 6
4	1552715_a_at	RXFP1	relaxin/insulin-like family peptide receptor 1
5	231804_at	RXFP1	relaxin/insulin-like family peptide receptor 1
6	206067_s_at	WT1	Wilms tumor 1
7	221275_s_at	MAGI3	membrane associated guanylate kinase, WW and PDZ domain containing 3
8	238206_at	RXFP1	relaxin/insulin-like family peptide receptor 1
9	1553962_s_at	RHOB	ras homolog gene family, member B
10	217373_x_at	MDM2	Mdm2 p53 binding protein homolog (mouse)
11	239309_at	DLX6	distal-less homeobox 6
12	214307_at	HGD	homogentisate 1,2-dioxygenase (homogentisate oxidase)
13	236911_at	RIMKLB	ribosomal modification protein rimK-like family member B
14	208126_s_at	CYP2C18	cytochrome P450, family 2, subfamily C, polypeptide 18
15	210717_at	UBE2D2	ubiquitin-conjugating enzyme E2D 2 (UBC4/5 homolog, yeast)
16	220017_x_at	CYP2C9	cytochrome P450, family 2, subfamily C, polypeptide 9
17	1553062_at	MOGAT1	monoacylglycerol O-acyltransferase 1
18	212567_s_at	MAP4	microtubule-associated protein 4
19	242394_at	CNKSR3	membrane associated guanylate kinase, WW and PDZ domain containing 1; CNKSR family member 3
20	213707_s_at	DLX5	distal-less homeobox 5
21	1554483_at	TMEM37	transmembrane protein 37
22	1566507_a_at	FBXO9	F-box protein 9
23	230964_at	FREM2	FRAS1 related extracellular matrix protein 2
24	216488_s_at	ATP11A	ATPase, class VI, type 11A
25	242870_at	RIMKLB	ribosomal modification protein rimK-like family member B
26	211214_s_at	DAPK1	death-associated protein kinase 1
27	219735_s_at	TFCP2L1	transcription factor CP2-like 1
28	33850_at	MAP4	microtubule-associated protein 4
29	225955_at	METRNL	meteorin, glial cell differentiation regulator-like; similar to meteorin, glial cell differentiation regulator-like
30	224209_s_at	GDA	guanine deaminase
31	216025_x_at	CYP2C9	cytochrome P450, family 2, subfamily C, polypeptide 9
32	206268_at	LEFTY1	left-right determination factor 1
33	207993_s_at	CHP	calcium binding protein P22
34	215103_at	CYP2C18	cytochrome P450, family 2, subfamily C, polypeptide 18
35	214421_x_at	CYP2C9	cytochrome P450, family 2, subfamily C, polypeptide 9
36	1563900_at	FAM83B	family with sequence similarity 83, member B
37	205095_s_at	ATP6V0A1	ATPase, H+ transporting, lysosomal V0 subunit a1
38	205221_at	HGD	homogentisate 1,2-dioxygenase (homogentisate oxidase)
39	216627_s_at	B4GALT1	UDP-Gal:betaGlcNAc beta 1,4- galactosyltransferase, polypeptide 1
40	213816_s_at	MET	met proto-oncogene (hepatocyte growth factor receptor)
41	225955_at	LOC653506	meteorin, glial cell differentiation regulator-like; similar to meteorin, glial cell differentiation regulator-like
42	244172_at	NPAS3	neuronal PAS domain protein 3
43	242394_at	MAGI1	membrane associated guanylate kinase, WW and PDZ domain containing 1; CNKSR family member 3
44	221011_s_at	LBH	limb bud and heart development homolog (mouse)
45	237737_at	LOC727770	similar to FLJ00310 protein
46	205799_s_at	SLC3A1	solute carrier family 3 (cystine, dibasic and neutral amino acid

			transporters, activator of cystine, dibasic and neutral amino acid transport), member 1
47	223958_s_at	DNAL1	dynein, axonemal, light chain 1
48	231826_at	RALGAPA2	chromosome 20 open reading frame 74
49	225224_at	C20orf112	chromosome 20 open reading frame 112
50	214443_at	PVR	poliovirus receptor
51	204457_s_at	GAS1	growth arrest-specific 1
52	215235_at	SPTAN1	spectrin, alpha, non-erythrocytic 1 (alpha-fodrin)
53	213582_at	ATP11A	ATPase, class VI, type 11A
54	227467_at	RDH10	retinol dehydrogenase 10 (all-trans)
55	235341_at	DNAJC3	DnaJ (Hsp40) homolog, subfamily C, member 3
56	221530_s_at	BHLHE41	basic helix-loop-helix family, member e41
57	214308_s_at	HGD	homogentisate 1,2-dioxygenase (homogentisate oxidase)
58	244056_at	SFTA2	surfactant associated 2
59	220087_at	BCMO1	beta-carotene 15,15'-monooxygenase 1
60	240935_at	HNF1B	HNF1 homeobox B
61	213807_x_at	MET	met proto-oncogene (hepatocyte growth factor receptor)
62	203853_s_at	GAB2	GRB2-associated binding protein 2
63	232882_at	FOXO1	forkhead box O1
64	1562969_at	DNMBP	dynamin binding protein
65	211986_at	AHNAK	AHNAK nucleoprotein
66	244276_at	KLB	klotho beta
67	242940_x_at	DLX6	distal-less homeobox 6
68	1556012_at	KLHDC7A	kelch domain containing 7A
69	200879_s_at	EPAS1	endothelial PAS domain protein 1
70	1552612_at	CDC42SE2	CDC42 small effector 2
71	203676_at	GNS	glucosamine (N-acetyl)-6-sulfatase
72	204682_at	LTBP2	latent transforming growth factor beta binding protein 2
73	216048_s_at	RHOBTB3	Rho-related BTB domain containing 3
74	237328_at	C14orf105	chromosome 14 open reading frame 105
75	222705_s_at	SLC25A15	solute carrier family 25 (mitochondrial carrier; ornithine transporter) member 15
76	211756_at	PTHLH	parathyroid hormone-like hormone
77	1559096_x_at	FBXO9	F-box protein 9
78	1558888_x_at	ZNF321	zinc finger protein 321
79	239381_at	KLK7	kallikrein-related peptidase 7
80	210301_at	XDH	xanthine dehydrogenase
81	242445_at	FGD4	FYVE, RhoGEF and PH domain containing 4
82	242776_at	ZCCHC6	zinc finger, CCHC domain containing 6
83	240139_at	UBE2D2	ubiquitin-conjugating enzyme E2D 2 (UBC4/5 homolog, yeast)
84	211020_at	GCNT2	glucosaminyl (N-acetyl) transferase 2, I-branching enzyme (I blood group)
85	201295_s_at	WSB1	WD repeat and SOCS box-containing 1
86	242774_at	SYNE2	spectrin repeat containing, nuclear envelope 2
87	208636_at	ACTN1	actinin, alpha 1
88	205921_s_at	SLC6A6	solute carrier family 6 (neurotransmitter transporter, taurine), member 6
89	1554375_a_at	NR1H4	nuclear receptor subfamily 1, group H, member 4
90	1570415_at	DDX52	DEAD (Asp-Glu-Ala-Asp) box polypeptide 52
91	222546_s_at	EPS8L2	EPS8-like 2
92	206300_s_at	PTHLH	parathyroid hormone-like hormone
