# Supplementary material for: Unsupervised analysis reveals two molecular subgroups of serous ovarian cancer with distinct gene expression profiles and survival
Source: J Cancer Res Clin Oncol. 2016 Mar 30;142(6):1239–52. doi: 10.1007/s00432-016-2147-y (PMC4869753; doi:10.1007/s00432-016-2147-y)
Supplement: Supplementary file 9 — Supplementary material 9 (PDF 46 kb) [file 432_2016_2147_MOESM9_ESM.pdf]

Supplementary Table 3.
Second mode of SVD done on all tumor samples (74 serous, 12 endometrioid, 9 clear cell and 6 undifferentiated ovarian cancers). 116 probesets. Second mode of SVD done on all tumors (101 ovarian cancer samples; 4 histological types: serous, undifferentiated, endometrioid, clear cell). This SVD mode contained 116 probe sets corresponding to 77 genes.

No	Affymetrix probe set	Gene symbol	Gene name
1	204222_s_at	GLIPR1	GLI pathogenesis-related 1
2	206584_at	LY96	lymphocyte antigen 96
3	202663_at	WIPF1	WAS/WASL interacting protein family, member 1
4	225763_at	RCSD1	RCSD domain containing 1
5	203603_s_at	ZEB2	zinc finger E-box binding homeobox 2
6	235593_at	ZEB2	zinc finger E-box binding homeobox 2
7	202664_at	WIPF1	WAS/WASL interacting protein family, member 1
8	219607_s_at	MS4A4A	membrane-spanning 4-domains, subfamily A, member 4
9	36030_at	IFFO1	intermediate filament family orphan 1
10	221210_s_at	NPL	N-acetylneuraminate pyruvate lyase (dihydrodipicolinate synthase)
11	217763_s_at	RAB31	RAB31, member RAS oncogene family
12	228964_at	PRDM1	PR domain containing 1, with ZNF domain
13	211668_s_at	PLAU	plasminogen activator, urokinase
14	209732_at	CLEC2B	C-type lectin domain family 2, member B
15	216950_s_at	FCGR1C	Fc fragment of IgG, high affinity Ic, receptor (CD64); Fc fragment of IgG, high affinity Ia, receptor (CD64)
16	217764_s_at	RAB31	RAB31, member RAS oncogene family
17	226136_at	GLIPR1	GLI pathogenesis-related 1
18	213416_at	ITGA4	integrin, alpha 4 (antigen CD49D, alpha 4 subunit of VLA-4 receptor)
19	204236_at	FLI1	Friend leukemia virus integration 1
20	1552316_a_at	GIMAP1	GTPase, IMAP family member 1
21	217762_s_at	RAB31	RAB31, member RAS oncogene family
22	206470_at	PLXNC1	plexin C1
23	205159_at	CSF2RB	colony stimulating factor 2 receptor, beta, low-affinity (granulocyte-macrophage)
24	213241_at	PLXNC1	plexin C1
25	212764_at	ZEB1	zinc finger E-box binding homeobox 1
26	205885_s_at	ITGA4	integrin, alpha 4 (antigen CD49D, alpha 4 subunit of VLA-4 receptor)
27	229560_at	TLR8	toll-like receptor 8
28	209621_s_at	PDLIM3	PDZ and LIM domain 3
29	235385_at	MARCH1	membrane-associated ring finger (C3HC4) 1
30	229367_s_at	GIMAP6	GTPase, IMAP family member 6
31	205269_at	LCP2	lymphocyte cytosolic protein 2 (SH2 domain containing leukocyte protein of 76kDa)
32	228438_at	LOC100132891	hypothetical protein LOC100132891
33	216950_s_at	FCGR1a	Fc fragment of IgG, high affinity Ic, receptor (CD64); Fc fragment of IgG, high affinity Ia, receptor (CD64)
34	220092_s_at	ANTXR1	anthrax toxin receptor 1
35	212588_at	PTPRC	protein tyrosine phosphatase, receptor type, C
36	202450_s_at	CTSK	cathepsin K
37	201108_s_at	THBS1	thrombospondin 1
38	219279_at	DOCK10	dedicator of cytokinesis 10
39	227140_at	INHBA	inhibin, beta A
40	204774_at	EVI2a	ecotropic viral integration site 2A
41	209721_s_at	IFFO1	intermediate filament family orphan 1
42	210895_s_at	CD86	CD86 molecule
43	201109_s_at	THBS1	thrombospondin 1
44	205479_s_at	PLAU	plasminogen activator, urokinase

45	212636_at	QKI	quaking homolog, KH domain RNA binding (mouse)
46	224356_x_at	MS4A6A	membrane-spanning 4-domains, subfamily A, member 6A
47	210644_s_at	LAIR1	leukocyte-associated immunoglobulin-like receptor 1
48	214770_at	MSR1	macrophage scavenger receptor 1
49	226142_at	GLIPR1	GLI pathogenesis-related 1
50	203561_at	FCGR2A	Fc fragment of IgG, low affinity IIa, receptor (CD32)
51	230550_at	MS4A6A	membrane-spanning 4-domains, subfamily A, member 6A
52	206796_at	WISP1	WNT1 inducible signaling pathway protein 1
53	204204_at	SLC31A2	solute carrier family 31 (copper transporters), member 2
54	226311_at	ADAMTS2	ADAM metallopeptidase with thrombospondin type 1 motif, 2
55	214085_x_at	GLIPR1	GLI pathogenesis-related 1
56	203868_s_at	VCAM1	vascular cell adhesion molecule 1
57	205796_at	TCP11L1	t-complex 11 (mouse)-like 1
58	225710_at	GNB4	guanine nucleotide binding protein (G protein), beta polypeptide 4
59	214511_x_at	FCGR1B	Fc fragment of IgG, high affinity Ib, receptor (CD64)
60	212464_s_at	FN1	fibronectin 1
61	1554966_a_at	FILIP1L	filamin A interacting protein 1-like
62	204463_s_at	EDNRA	endothelin receptor type A
63	216442_x_at	FN1	fibronectin 1
64	201279_s_at	DAB2	disabled homolog 2, mitogen-responsive phosphoprotein (Drosophila)
65	235821_at	WISP1	WNT1 inducible signaling pathway protein 1
66	211571_s_at	VCAN	versican
67	228071_at	GIMAP7	GTPase, IMAP family member 7
68	202765_s_at	FBN1	fibrillin 1
69	219777_at	GIMAP6	GTPase, IMAP family member 6
70	206715_at	TFEC	transcription factor EC
71	219892_at	TN6SF1	transmembrane 6 superfamily member 1
72	207238_s_at	PTPRC	protein tyrosine phosphatase, receptor type, C
73	215617_at	SPATS2L	spermatogenesis associated, serine-rich 2-like
74	206710_s_at	EPB41L3	erythrocyte membrane protein band 4.1-like 3
75	227266_s_at	FYB	FYN binding protein (FYB-120/130)
76	203473_at	SLCO2B1	solute carrier organic anion transporter family, member 2B1
77	207691_x_at	ENTPD1	ectonucleoside triphosphate diphosphohydrolase 1
78	210495_x_at	FN1	fibronectin 1
79	215646_s_at	VCAN	versican
80	211776_s_at	EPB41L3	erythrocyte membrane protein band 4.1-like 3
81	225464_at	FRMD6	FERM domain containing 6
82	201278_at	DAB2	disabled homolog 2, mitogen-responsive phosphoprotein (Drosophila)
83	212587_s_at	Ptprc	protein tyrosine phosphatase, receptor type, C
84	201852_x_at	COL3A1	collagen, type III, alpha 1
85	218231_at	NAGK	N-acetylglucosamine kinase
86	203416_at	Cd53	CD53 molecule
87	205686_s_at	CD86	CD86 molecule
88	204464_s_at	EDNRA	endothelin receptor type A
89	226219_at	ARHGAP30	Rho GTPase activating protein 30
90	219243_at	GIMAP4	GTPase, IMAP family member 4
91	209474_s_at	ENTPD1	ectonucleoside triphosphate diphosphohydrolase 1
92	226834_at	ASAM	adipocyte-specific adhesion molecule
93	204221_x_at	GLIPR1	GLI pathogenesis-related 1
94	203083_at	THBS2	thrombospondin 2
95	219574_at	MARCH1	membrane-associated ring finger (C3HC4) 1
96	202766_s_at	FBN1	fibrillin 1
97	227346_at	IKFZ1	IKAROS family zinc finger 1 (Ikaros)
98	219947_at	CLEC4A	C-type lectin domain family 4, member A
99	211795_s_at	FYB	FYN binding protein (FYB-120/130)

100	201792_at	AEBP1	AE binding protein 1
101	218196_at	OSTM1	osteopetrosis associated transmembrane protein 1
102	211813_x_at	DCN	decorin
103	205884_at	ITGA4	integrin, alpha 4 (antigen CD49D, alpha 4 subunit of VLA-4 receptor)
104	211719_x_at	FN1	fibronectin 1
105	213125_at	OLFML2B	olfactomedin-like 2B
106	221730_at	COL5A2	collagen, type V, alpha 2
107	202665_s_at	WIPF1	WAS/WASL interacting protein family, member 1
108	213139_at	SNAI2	snail homolog 2 (Drosophila)
109	224927_at	KIAA1949	KIAA1949
110	204220_at	GMFG	glia maturation factor, gamma
111	201616_s_at	CALD1	caldesmon 1
112	211896_s_at	DCN	decorin
113	229554_at	LUM	lumican
114	238668_at	NCKAP1L	NCK-associated protein 1-like
115	204006_s_at	FCGR3A	Fc fragment of IgG, low affinity IIIa, receptor (CD16a)
116	212414_s_at	SEPT6	septin 6
