# Supplementary material for: Unsupervised analysis reveals two molecular subgroups of serous ovarian cancer with distinct gene expression profiles and survival
Source: J Cancer Res Clin Oncol. 2016 Mar 30;142(6):1239–52. doi: 10.1007/s00432-016-2147-y (PMC4869753; doi:10.1007/s00432-016-2147-y)
Supplement: Supplementary file 11 — Supplementary material 11 (PDF 67 kb) [file 432_2016_2147_MOESM11_ESM.pdf]

Supplementary Table 5.

Significant signaling pathways and gene sets that may be affected by changed expression of genes connected with two clusters with distinct overall survival.

GSEA analysis was done on all genes connected with two clusters, using c2: curated gene set collections from MSigDB (3724 gene sets). We applied two independent tests: the LS permutation test and Efron-Tibshirani GSA test to identify gene sets differentiating two clusters of ovarian cancer samples. LS permutation test finds gene sets which have more genes differentially expressed among the classes than expected by chance. Efron-Tibshirani's test uses 'maxmean' statistics to identify gene sets differentially expressed.

We considered a GSEA category significantly differentially regulated if significance level in either of the tests was less than 0.05 after Benjamini-Hochberg False Discovery Rate (FDR) multiple test correction.

No	Gene set / Signaling pathway	No of genes	FDR corrected LS permutation p-value	FDR corrected Efron-Tibshirani's GSA test p-value
1	ALONSO_METASTASIS_EMT_UP	82	0,00005	< 0.005 (+)
2	ANASTASSIOU_CANCER_MESENCHYMAL_TRANSITION_SIGNATURE	161	0,00005	< 0.005 (+)
3	AZARE_NEOPLASTIC_TRANSFORMATION_BY_STAT3_DN	336	0,00005	< 0.005 (+)
4	BEGUM_TARGETS_OF_PAX3_FOXO1_FUSION_AND_PAX3	25	0,00005	< 0.005 (+)
5	BEGUM_TARGETS_OF_PAX3_FOXO1_FUSION_DN	111	0,00005	< 0.005 (+)
6	BHATI_G2M_ARREST_BY_2METHOXYESTRADIOL_DN	238	0,00005	< 0.005 (-)
7	BIOCARTA_CTL_PATHWAY	26	0,00005	0.05783 (+)
8	BIOCARTA_FIBRINOLYSIS_PATHWAY	21	0,00005	< 0.005 (+)
9	BIOCARTA_LYM_PATHWAY	29	0,00005	0.05783 (+)
10	BIOCARTA_TCRA_PATHWAY	20	0,00005	0.05783 (+)
11	BIOCARTA_TCYTOTOXIC_PATHWAY	25	0,00005	0.05783 (+)
12	BOQUEST_STEM_CELL_UP	623	0,00005	< 0.005 (+)
13	BRUECKNER_TARGETS_OF_MIRLET7A3_DN	183	0,00005	< 0.005 (+)
14	CHANG_POU5F1_TARGETS_UP	44	0,00005	< 0.005 (+)
15	CHARAFE_BREAST_CANCER_BASAL_VS_MESENCHYMAL_DN	123	0,00005	< 0.005 (+)
16	CHIANG_LIVER_CANCER_SUBCLASS_CTNNB1_DN	345	0,00005	< 0.005 (+)
17	CHIBA_RESPONSE_TO_TSA_UP	120	0,00005	< 0.005 (+)
18	CLAUS_PGR_POSITIVE_MENINGIOMA_DN	42	0,00005	< 0.005 (+)
19	CORRE_MULTIPLE_MYELOMA_DN	141	0,00005	< 0.005 (+)
20	COWLING_MYCN_TARGETS	88	0,00005	< 0.005 (+)
21	CREIGHTON_ENDOCRINE_THERAPY_RESISTANCE_4	697	0,00005	< 0.005 (-)

22	CROMER_TUMORIGENESIS_UP	137	0,00005	< 0.005 (+)
23	CROONQUIST_NRAS_VS_STROMAL_STIMULATION_DN	213	0,00005	< 0.005 (+)
24	CROONQUIST_STROMAL_STIMULATION_UP	148	0,00005	< 0.005 (+)
25	DANG_REGULATED_BY_MYC_DN	691	0,00005	< 0.005 (+)
26	DASU_IL6_SIGNALING_DN	22	0,00005	< 0.005 (+)
27	DASU_IL6_SIGNALING_SCAR_DN	45	0,00005	< 0.005 (+)
28	DAVICIONI_RHABDOMYOSARCOMA_PAX_FOXO1_FUSION_DN	41	0,00005	< 0.005 (+)
29	DAVICIONI_TARGETS_OF_PAX_FOXO1_FUSIONS_DN	162	0,00005	< 0.005 (+)
30	DELYS_THYROID_CANCER_UP	996	0,00005	< 0.005 (+)
31	DUNNE_TARGETS_OF_AML1_MTG8_FUSION_DN	56	0,00005	< 0.005 (+)
32	ELVIDGE_HIF2A_TARGETS_UP	16	0,00005	< 0.005 (+)
33	FARMER_BREAST_CANCER_CLUSTER_5	42	0,00005	< 0.005 (+)
34	FLOTHO_PEDIATRIC_ALL_THERAPY_RESPONSE_UP	128	0,00005	< 0.005 (-)
35	FRIDMAN_SENESCENCE_DN	28	0,00005	< 0.005 (+)
36	FRIDMAN_SENESCENCE_UP	203	0,00005	< 0.005 (+)
37	GILDEA_METASTASIS	85	0,00005	< 0.005 (+)
38	GRANDVAUX_IRF3_TARGETS_DN	59	0,00005	< 0.005 (+)
39	GRUETZMANN_PANCREATIC_CANCER_UP	887	0,00005	< 0.005 (+)
40	GU_PDEF_TARGETS_UP	214	0,00005	< 0.005 (+)
41	HADDAD_T_LYMPHOCYTE_AND_NK_PROGENITOR_DN	128	0,00005	< 0.005 (+)
42	HAEGERSTRAND_RESPONSE_TO_IMATINIB	24	0,00005	< 0.005 (+)
43	HAHTOLA_CTCL_PATHOGENESIS	29	0,00005	< 0.005 (+)
44	HERNANDEZ_ABERRANT_MITOSIS_BY_DOCETACEL_2NM_UP	209	0,00005	< 0.005 (+)
45	HERNANDEZ_ABERRANT_MITOSIS_BY_DOCETACEL_4NM_UP	47	0,00005	< 0.005 (+)
46	HERNANDEZ_MITOTIC_ARREST_BY_DOCETAXEL_2_DN	45	0,00005	< 0.005 (+)
47	HERNANDEZ_MITOTIC_ARREST_BY_DOCETAXEL_2_UP	142	0,00005	< 0.005 (+)
48	HOSHIDA_LIVER_CANCER_LATE_RECURRENCE_UP	164	0,00005	< 0.005 (+)
49	HOSHIDA_LIVER_CANCER_SUBCLASS_S1	552	0,00005	< 0.005 (+)
50	HOSHIDA_LIVER_CANCER_SURVIVAL_UP	195	0,00005	< 0.005 (+)
51	JAEGER_METASTASIS_UP	115	0,00005	< 0.005 (+)
52	KARAKAS_TGFB1_SIGNALING	44	0,00005	< 0.005 (+)
53	KEGG_ECM_RECEPTOR_INTERACTION	235	0,00005	< 0.005 (+)
54	KEGG_FOCAL_ADHESION	595	0,00005	0.05783 (+)

55	KEGG_RIBOSOME	144	0,00005	< 0.005 (-)
56	KOYAMA_SEMA3B_TARGETS_DN	924	0,00005	< 0.005 (-)
57	KYNG_DNA_DAMAGE_DN	522	0,00005	< 0.005 (+)
58	LI_AMPLIFIED_IN_LUNG_CANCER	369	0,00005	< 0.005 (-)
59	LI_WILMS_TUMOR_VS_FETAL_KIDNEY_2_DN	145	0,00005	< 0.005 (+)
60	LIANG_SILENCED_BY_METHYLATION_UP	65	0,00005	< 0.005 (+)
61	LIEN_BREAST_CARCINOMA_METAPLASTIC	107	0,00005	< 0.005 (+)
62	LINDGREN_BLADDER_CANCER_CLUSTER_2B	959	0,00005	< 0.005 (+)
63	LINDGREN_BLADDER_CANCER_HIGH_RECURRENCE	137	0,00005	< 0.005 (+)
64	LINDVALL_IMMORTALIZED_BY_TERT_DN	217	0,00005	< 0.005 (+)
65	LIU_TARGETS_OF_VMYB_VS_CMYB_DN	110	0,00005	< 0.005 (+)
66	LU_TUMOR_ENDOTHELIAL_MARKERS_UP	52	0,00005	< 0.005 (+)
67	LU_TUMOR_VASCULATURE_UP	64	0,00005	< 0.005 (+)
68	LUCAS_HNF4A_TARGETS_DN	22	0,00005	< 0.005 (+)
69	MAHAJAN_RESPONSE_TO_IL1A_DN	210	0,00005	< 0.005 (+)
70	MATTIOLI_MULTIPLE_MYELOMA_WITH_14Q32_TRANSLOCATIONS	96	0,00005	< 0.005 (+)
71	MIKKELSEN_MEF_LCP_WITH_H3K4ME3	267	0,00005	< 0.005 (+)
72	MIPS_60S_RIBOSOMAL_SUBUNIT_CYTOPLASMIC	78	0,00005	< 0.005 (-)
73	MIPS_NOP56P_ASSOCIATED_PRE_RRNA_COMPLEX	218	0,00005	< 0.005 (-)
74	MIPS_RIBOSOME_CYTOPLASMIC	136	0,00005	< 0.005 (-)
75	MISHRA_CARCINOMA_ASSOCIATED_FIBROBLAST_UP	51	0,00005	< 0.005 (+)
76	MIYAGAWA_TARGETS_OF_EWSR1_ETS_FUSIONS_DN	604	0,00005	< 0.005 (+)
77	MOROSETTI_FACIOSCAPULOHUMERAL_MUSCULAR_DISTROPHY_UP	37	0,00005	< 0.005 (+)
78	MULLIGHAN_MLL_SIGNATURE_1_DN	611	0,00005	< 0.005 (-)
79	NAKAMURA_ADIPOGENESIS_EARLY_DN	114	0,00005	< 0.005 (+)
80	NAKAMURA_ADIPOGENESIS_LATE_DN	111	0,00005	< 0.005 (+)
81	NAKAMURA_CANCER_MICROENVIRONMENT_UP	65	0,00005	< 0.005 (+)
82	NAKAMURA_TUMOR_ZONE_PERIPHERAL_VS_CENTRAL_UP	725	0,00005	< 0.005 (+)
83	NEWMAN_ERCC6_TARGETS_DN	107	0,00005	< 0.005 (+)
84	NIELSEN_GIST_AND_SYNOVIAL_SARCOMA_DN	42	0,00005	< 0.005 (+)
85	NIELSEN_MALIGNAT_FIBROUS_HISTIOCYTOMA_UP	25	0,00005	< 0.005 (+)
86	OHM_EMBRYONIC_CARCINOMA_DN	22	0,00005	< 0.005 (+)
87	OHM_METHYLATED_IN_ADULT_CANCERS	74	0,00005	< 0.005 (+)

88	OLSSON_E2F3_TARGETS_DN	100	0,00005	< 0.005 (+)
89	ONDER_CDH1_SIGNALING_VIA_CTNNB1	202	0,00005	< 0.005 (+)
90	ONDER_CDH1_TARGETS_2_UP	671	0,00005	< 0.005 (+)
91	OXFORD_RALA_AND_RALB_TARGETS_DN	23	0,00005	< 0.005 (+)
92	OXFORD_RALB_TARGETS_UP	23	0,00005	< 0.005 (+)
93	OZANNE_AP1_TARGETS_DN	21	0,00005	< 0.005 (+)
94	PETRETTO_CARDIAC_HYPERTROPHY	115	0,00005	< 0.005 (+)
95	PETROVA_PROX1_TARGETS_DN	154	0,00005	< 0.005 (+)
96	PICCALUGA_ANGIOIMMUNOBLASTIC_LYMPHOMA_UP	558	0,00005	< 0.005 (+)
97	PID_AVB3_INTEGRIN_PATHWAY	227	0,00005	< 0.005 (+)
98	PID_FRA_PATHWAY	95	0,00005	< 0.005 (+)
99	REACTOME_3_UTR_MEDIATED_TRANSLATIONAL_REGULATION	193	0,00005	< 0.005 (-)
100	REACTOME_A_TETRASACCHARIDE_LINKER_SEQUENCE_IS_REQUIRED_FOR_GAG_SYNTHESIS	58	0,00005	< 0.005 (+)
101	REACTOME_CELL_SURFACE_INTERACTIONS_AT_THE_VASCULAR_WALL	223	0,00005	0.05783 (+)
102	REACTOME_CHONDROITIN_SULFATE_BIOSYNTHESIS	45	0,00005	< 0.005 (+)
103	REACTOME_CHONDROITIN_SULFATE_DERMATAN_SULFATE_METABOLISM	111	0,00005	< 0.005 (+)
104	REACTOME_COLLAGEN_FORMATION	136	0,00005	< 0.005 (+)
105	REACTOME_CS_DS_DEGRADATION	42	0,00005	< 0.005 (+)
106	REACTOME_DEGRADATION_OF_THE_EXTRACELLULAR_MATRIX	58	0,00005	< 0.005 (+)
107	REACTOME_EXTRACELLULAR_MATRIX_ORGANIZATION	194	0,00005	< 0.005 (+)
108	REACTOME_GLYCOSAMINOGLYCAN_METABOLISM	257	0,00005	< 0.005 (+)
109	REACTOME_HEPARAN_SULFATE_HEPARIN_HS_GAG_METABOLISM	117	0,00005	0.05783 (+)
110	REACTOME_INFLUENZA_VIRAL_RNA_TRANSCRIPTION_AND_REPLICATION	183	0,00005	< 0.005 (-)
111	REACTOME_INTEGRIN_CELL_SURFACE_INTERACTIONS	222	0,00005	< 0.005 (+)
112	REACTOME_KERATAN_SULFATE_DEGRADATION	23	0,00005	0.05783 (+)
113	REACTOME_METABOLISM_OF_PROTEINS	857	0,00005	< 0.005 (-)
114	REACTOME_NONSENSE_MEDIATED_DECAY_ENHANCED_BY_THE_EXON_JUNCTION_COMPLEX	187	0,00005	< 0.005 (-)
115	REACTOME_PEPTIDE_CHAIN_ELONGATION	148	0,00005	< 0.005 (-)
116	REACTOME_PLATELET_ADHESION_TO_EXPOSED_COLLAGEN	31	0,00005	< 0.005 (+)
117	REACTOME_SIGNALING_BY_PDGF	340	0,00005	0.05783 (+)

118	REACTOME_SRP_DEPENDENT_COTRANSLATIONAL_PROTEIN_TARGETING_TO_MEMBRANE	190	0,00005	< 0.005 (-)
119	BIOCARTA_CARDIACEGF_PATHWAY	54	0,00012	0.05783 (+)
120	REACTOME_TRANSLATION	279	0,00025	< 0.005 (-)
121	REACTOME_INFLUENZA_LIFE_CYCLE	276	0,0038	< 0.005 (-)
122	REACTOME_GLYCOPROTEIN_HORMONES	13	0,00809	0.05783 (+)
123	REACTOME_FORMATION_OF_THE_TERNARY_COMPLEX_AND_SUBSEQUENTLY_THE_43S_COMPLEX	97	0,01713	< 0.005 (-)
124	REACTOME_ACTIVATION_OF_THE_MRNA_UPON_BINDING_OF_THE_CAP_BINDING_COMPLEX_AND_EIFS_AND_SUBSEQUENT_BINDING_TO_43S	113	0,02187	< 0.005 (-)
125	REACTOME_FORMATION_OF_ATP_BY_CHEMIOSMOTIC_COUPLING	30	0,03646	< 0.005 (-)
